# Engineering Resatorvid‐Loaded Sub‐Microgels of Epigallocatechin‐3‐gallate/Hyaluronic Acid to Treat Acute Lung Injury

**DOI:** 10.1002/adhm.202505916

**Published:** 2026-03-24

**Authors:** Bo Liu, Olawale Alimi Alimi, Benjamin Stearnes, Svetlana Romanova, Brady J. Sillman, Mena Asha Krishnan, Kristina Bailey, Benson J. Edagwa, Huanhuan Joyce Chen, Han‐jun Wang, Bin Duan

**Affiliations:** ^1^ Mary & Dick Holland Regenerative Medicine Program University of Nebraska Medical Center Omaha Nebraska USA; ^2^ Division of Cardiovascular Medicine Department of Internal Medicine University of Nebraska Medical Center Omaha Nebraska USA; ^3^ Department of Pharmaceutical Sciences University of Nebraska Medical Center Omaha Nebraska USA; ^4^ Department of Pharmacology and Experimental Neuroscience University of Nebraska Medical Center Omaha Nebraska USA; ^5^ Division of Pulmonary, Critical Care & Sleep Medicine Department of Internal Medicine University of Nebraska Medical Center Omaha Nebraska USA; ^6^ Department of Medicine VA Nebraska Western Iowa Healthcare System Omaha Nebraska USA; ^7^ Pritzker School of Molecular Engineering University of Chicago Chicago Illinois USA; ^8^ Ben May Department for Cancer Research University of Chicago Chicago Illinois USA; ^9^ Department of Anesthesiology University of Nebraska Medical Center Omaha Nebraska USA; ^10^ Department of Surgery University of Nebraska Medical Center Omaha Nebraska USA; ^11^ Department of Mechanical and Materials Engineering University of Nebraska Lincoln Lincoln Nebraska USA

**Keywords:** acute lung injury, anti‐inflammatory, anti‐oxidation, resatorvid, sub‐microgel

## Abstract

Acute lung injury (ALI) and acute respiratory distress syndrome (ARDS) remain life‐threatening conditions with high morbidity and mortality, largely due to excessive reactive oxygen species (ROS) generation and uncontrolled inflammatory cascades. Despite extensive pharmacological investigations, effective and targeted therapies are still lacking. This study develops an innovative hyaluronic acid (HA)‐epigallocatechin gallate (EGCG)‐amino phenylboronic acid (PBA) based sub‐microgel (HAEP sub‐microgel) platform capable of integrating multiple dynamic interactions (esterification, boronic ester bonds, and hydrogen bonding) to achieve stability, lung delivery, and antioxidant activity. The HAEP sub‐microgel system is employed to deliver the anti‐inflammatory agent resatorvid (Res) to the injured lung in a bleomycin‐induced ALI mouse model. The HAEP@Res sub‐microgels (0.1–1.4 µm) exhibit excellent biocompatibility, effectively scavenge intracellular ROS in human lung fibroblasts in vitro. Moreover, the HAEP@Res intratracheal administration significantly reduces histopathological lung tissue injury, and suppresses pro‐inflammatory cytokine secretion (TNF‐α, IL‐6, IL‐1β) and gene expression (*Tlr4*, *Tnf*, *Il6*, *Il1b*, *Nrf2*, *Nos2*, *Tgfb*, *Ccl2*) in vivo. Altogether, this study establishes a versatile HAEP sub‐microgel‐based drug delivery system for anti‐inflammatory payloads, effectively alleviating lung inflammation and promoting ALI recovery, thereby demonstrating therapeutic potential for ALI/ARDS and other inflammation‐related diseases.

## Graphical abstract

This study presents HAEP@Res sub‐microgels as a lung‐targeted delivery system integrating antioxidant activity with anti‐inflammatory therapy. The sub‐microgels demonstrate excellent biocompatibility, efficiently scavenge intracellular ROS, and downregulate pro‐inflammatory cytokines and genes in a Bleo‐induced ALI mouse model. These findings highlight HAEP@Res as a versatile platform with therapeutic potential for ALI/ARDS and other inflammation‐driven diseases.

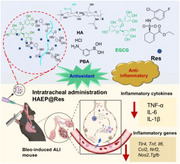

## 1 Introduction

Acute lung injury (ALI) and acute respiratory distress syndrome (ARDS) are severe clinical syndromes characterized by disruption of the alveolar‐capillary barrier, leading to non‐cardiogenic pulmonary edema, ultimately leading to refractory hypoxemia and substantial mortality [1, 2]. ALI/ARDS are frequent complications in critically ill patients and can be precipitated by diverse etiologies, including sepsis, pneumonia, aspiration, and trauma [3, 4, 5]. Disease progression is associated with exacerbation of hypoxemia and an uncontrolled inflammatory response, often culminating in severe respiratory failure and poor clinical outcomes [4, 6, 7]. The incidence of ALI/ARDS is estimated at approximately 200 000 cases per year in the United States alone, and a mortality of 40% worldwide [2, 8, 9]. Despite advances in mechanical ventilation and supportive treatments, the mortality rate and prognoses remain poor [10, 11]. Moreover, there are still no specific pharmacological treatments that effectively reduce mortality of patients with ALI/ARDS [12]. Therefore, there is an urgent need to develop safe and effective therapeutic strategies for ALI/ARDS therapy by modulating the inflammatory disorder and suppressing the production of reactive oxygen species (ROS) and inflammatory cytokines.

Pulmonary delivery of therapeutic agents has gained significant attention in recent years due to its ability to enhanced local drug concentration while minimizing systemic toxicity [13, 14]. Among various pulmonary delivery strategies, polymeric vehicles have emerged as particularly attractive platforms because they enable precise control over physicochemical properties, including size, shape, architecture, charge, and surface functionality [15, 16]. A wide range of polymeric drug delivery systems are composed of biodegradable and biocompatible synthetic polymers, such as nanoparticles, polymeric micelles, microparticles, and dendrimers [13, 16, 17]. These delivery systems are capable of transporting both hydrophilic and hydrophobic drugs to specific locations within the lungs, maintaining therapeutic concentrations for extended periods, reducing dosing frequency, and ultimately improving treatment outcomes in pulmonary diseases [15, 18]. In addition to increasing solubility, dissolution, and bioavailability, polymeric carriers can also decrease drug toxicity and prolong the local presence of therapeutic agents at the target site [19]. Despite these advantages, the effectiveness of polymeric drug carriers in pulmonary delivery is limited by physiological barriers within the respiratory tract and the highly dynamic lung environment [20]. These limitations are further exacerbated in ALI/ARDS, where complex inflammatory signaling cascades and the destruction of biological barriers present substantial obstacles to the drug delivery‐based therapies [21]. Therefore, the delivery system needs to be able to maintain stability to prevent possible mucus adhesion, blockage, or interference with drug release. The delivery system should be designed to achieve the key requirements of effective drug delivery: 1) avoid mucociliary clearance and achieve deep lung delivery with suitable size [13]; 2) avoid rapid alveolar macrophage clearance [22]; 3) facilitate sustained drug release [23].

Among the polymeric vehicles, hyaluronic acid (HA)‐based vehicles have been widely studied due to their favorable properties, including biocompatibility, biodegradability, low immunogenicity, and bioactivity, which is a requirement for pulmonary drug delivery systems [24, 25]. Epigallocatechin gallate (EGCG), a Food and Drug Administration (FDA) approved polyphenol from green tea that can scavenge reactive oxygen species and prevent inflammation‐induced oxidative damage, which can regulate inflammatory and oxidative stress in ALI [26, 27]. In addition, Toll‐like receptor 4 (TLR4) has been recognized as a key innate immune receptor broadly expressed in vivo that detects pathogen‐associated molecular patterns and whose dysregulation contributes to the development of various diseases, including pulmonary disease, rheumatoid arthritis, and cardiovascular diseases [28, 29]. In lung disease, TLR4 plays a critical role in facilitating interactions between innate immune cells and lung parenchymal cells [30, 31, 32]. Therefore, targeting TLR4 may be an effective therapeutic strategy for managing bleomycin (Bleo)‐induced lung injury. For instance, resatorvid (Res) is a small‐molecule, selective TLR4 inhibitor, and shows promising results in murine models of lipopolysaccharide (LPS), virus or acute cigarette smoke‐induced pulmonary inflammation [33, 34, 35].

**SCHEME 1:**
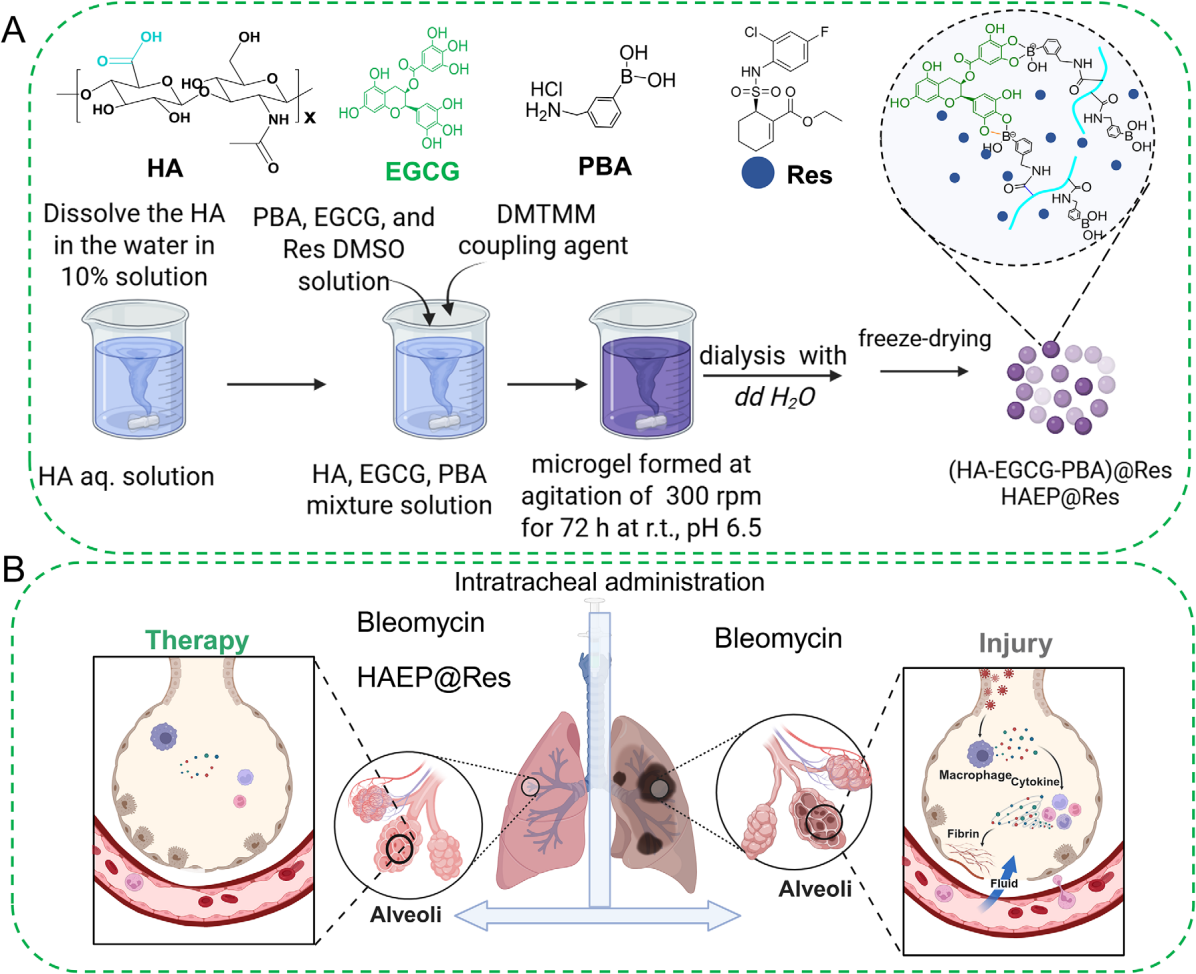
Schematic illustration of the fabrication of HAEP@Res sub‐microgels and their therapeutic application in a Bleo‐induced lung injury mouse model. (A) HAEP@Res sub‐microgels were fabricated through conjugation among HA, EGCG, and PBA, with Res encapsulated simultaneously during the reaction. (B) After Bleo administration, HAEP@Res sub‐microgels were intratracheally delivered into the lung to promote the repair of the injured lung. The schematic was created with BioRender.com under an authorized license.

In our current study, we developed sub‐microgels (HAEP) via a one‐pot procedure by conjugating the amino groups of 3‐aminophenylboronic acid (PBA) with the carboxyl groups of HA with the addition of the coupling reagent 4‐(4,6‐dimethoxy‐1,3,5‐triazin‐2‐yl)‐4‐methylmorpholinium chloride (DMTMM), while simultaneously forming boronic ester dynamic covalent bonds between EGCG and PBA (Scheme 1A). We further encapsulated the drug Res into HAEP sub‐microgels (denoted as HAEP@Res) as a delivery platform to treat murine Bleo‐induced ALI (Scheme 1B). We then analyzed the physiochemical properties, examined the biocompatibility, and antioxidant effects in primary human lung fibroblasts (HLF) in vitro. Finally, we evaluated the in vivo efficacy of HAEP@Res sub‐microgels for ALI repair, demonstrating that the HAEP sub‐microgel‐based drug delivery of Res effectively alleviates lung inflammation and promotes ALI recovery.

## 2 Results

### 2.1 HAEP@Res Sub‐Microgels Preparation and Characterization

**FIGURE 1:**
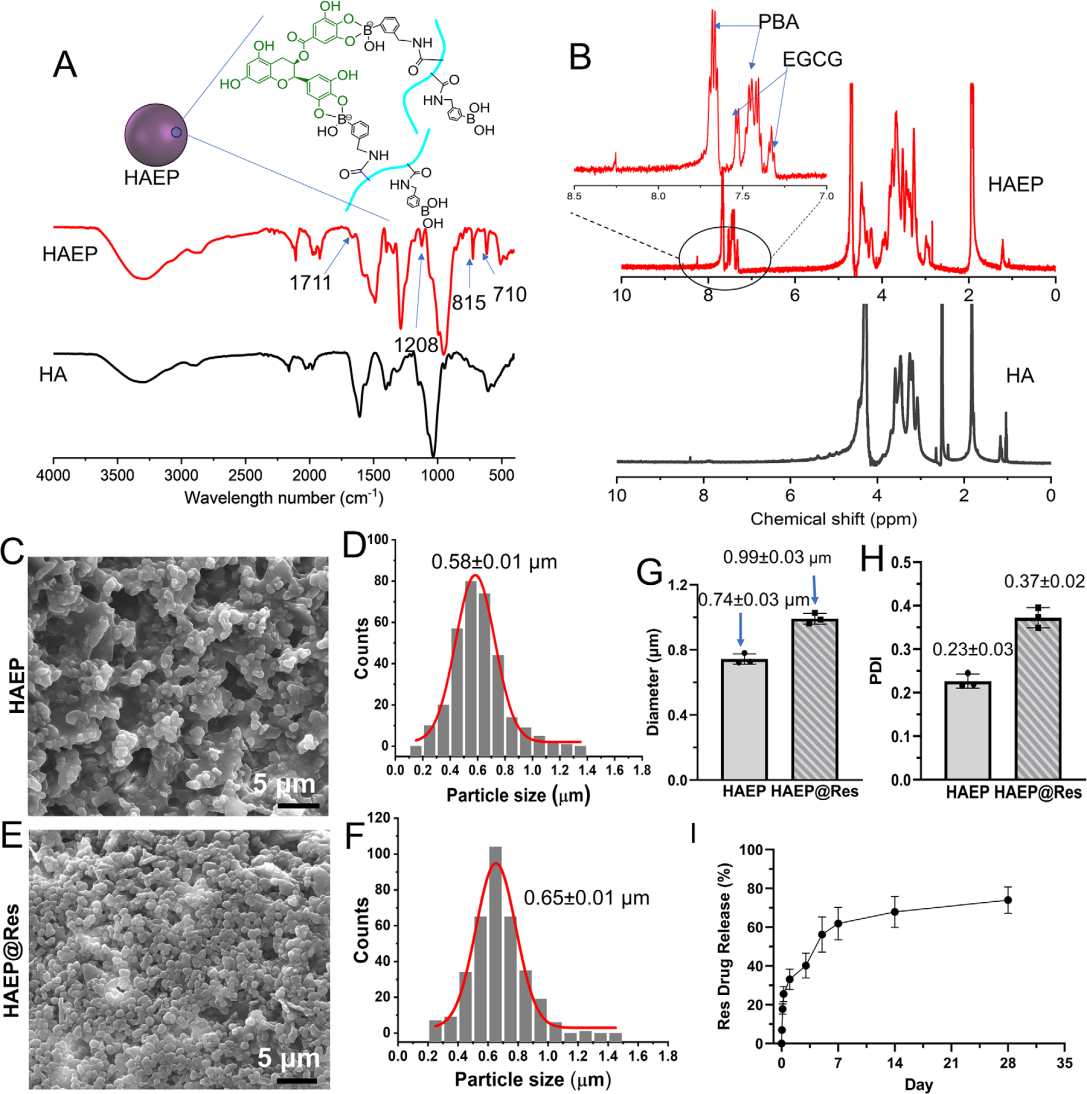
Characterization of HAEP sub‐microgels. (A) FTIR spectra of HAEP; (B) ^1^H NMR spectra of HAEP; SEM images (C, E) and size and distribution (D, F) of HAEP and HAEP@Res sub‐microgels; DLS results showing the sizes (G) and PDI (H) of sub‐microgels. (I) The accumulative Res drug release profile within 28 d (*n* = 4).

In a typical experiment as shown in Scheme 1A, a one‐pot synthesis approach was employed to produce HAEP sub‐microgels and encapsulate hydrophobic Res drug within these sub‐microgels. This synthesis was achieved by pre‐mixed HA aqueous solution with EGCG, PBA, and Res DMSO organic solution, and followed by the addition of the DMTMM to initiate the self‐assembly process by a conjugation reaction among HA, EGCG, and PBA, resulting in the formation of Res‐loaded HAEP sub‐microgel emulsion‐like solution. The HAEP sub‐microgels were verified according to the FTIR and ^1^H NMR spectra (Figure 1A,B). In the FTIR spectra (Figure 1A), there were obvious characteristic peaks of PBA at 815 and 710 cm^−1^ (Ar─H deformation vibration of the aromatic ring). The peaks at 1521–1429 cm^−1^ were attributed to aromatic ring deformation [36]. In addition, HAEP exhibited new peaks at 1711 and 1208 cm^−1^, which were assigned to ester C = O stretching vibrations and aromatic C─O stretch of EGCG residues [37, 38]. In the ^1^H NMR spectra of HAEP (Figure 1B), the CH groups in the aromatic ring structures of EGCG are labeled by arrows (*δ* = 7.61 ppm, *δ* = 7.21 ppm) [39]. The CH groups in the aromatic ring structures of PBA residues are marked by arrows (δ, 7.78–7.73 ppm and 7.44–7.34 ppm. Based on the special signal peaks of PBA, EGCG, and HA, the actual molar percentages of PBA and EGCG were calculated as 21.2% and 18.6%, respectively.

We further evaluated the particle size and morphology of sub‐microgels by using scanning electron microscopy (SEM) and dynamic light scattering (DLS) methods (Figure 1C–H). As shown in Figure 1C,E, the HAEP and HAEP@Res exhibited a regular spherical shape in dry states. The HAEP sub‐microgels might be formed and stabilized by hydrogen bonding, hydrophobic interactions, and π–π stacking between EGCG and PBA molecules [40]. The particle size of HAEP@Res is around 0.65 ± 0.01 µm, which is slightly larger than HAEP (around 0.58 ± 0.01 µm) due to encapsulation of Res drug (Figure 1D,F). Additionally, the HAEP sub‐microgels can be dispersed in water directly. DLS analysis revealed the particle size and distribution for HAEP were 0.74 ± 0.03 µm and 0.23 ± 0.03, respectively (Figure 1G). As expected, the particle size and distribution for HAEP@Res sub‐microgels was 0.99 ± 0.03 µm and 0.37 ± 0.02, respectively, which were slightly larger than HAEP (Figure 1H). Interestingly, the particle sizes of both HAEP and HAEP@Res were smaller in the dry state than in aqueous solution. The observed enlargement upon hydration might result from the swelling behavior of hydrogel‐derived, sub‐microgel particles [41]. Collectively, these findings confirm the successful formulation and stability of HAEP sub‐microgels, positioning them as a highly promising platform for the local drug delivery applications.

High‐performance liquid chromatography (HPLC) analysis showed that the final loading efficiency (LE) of Res in HAEP@Res sub‐microgels was approximately 5% (w/w), with an encapsulation efficiency (EE) of Res drug around 83% (w/w). Furthermore, the release of Res from the HAEP@Res sub‐microgels were calculated based on the standard curve (Figure S1). As shown in Figure 1I, HAEP@Res sub‐microgels exhibited slow and sustained release behaviors over 28 days. The release profile of Res showed about 33% burst releases and 79% cumulative drug release of Res over this period. These results demonstrated that the HAEP@Res sub‐microgels, with high EE of Res, can be fabricated using a facile approach, offering great potential for localized therapeutic delivery in ALI.

### 2.2 Cytocompatibility of HAEP@Res Sub‐Microgels In Vitro

**FIGURE 2:**
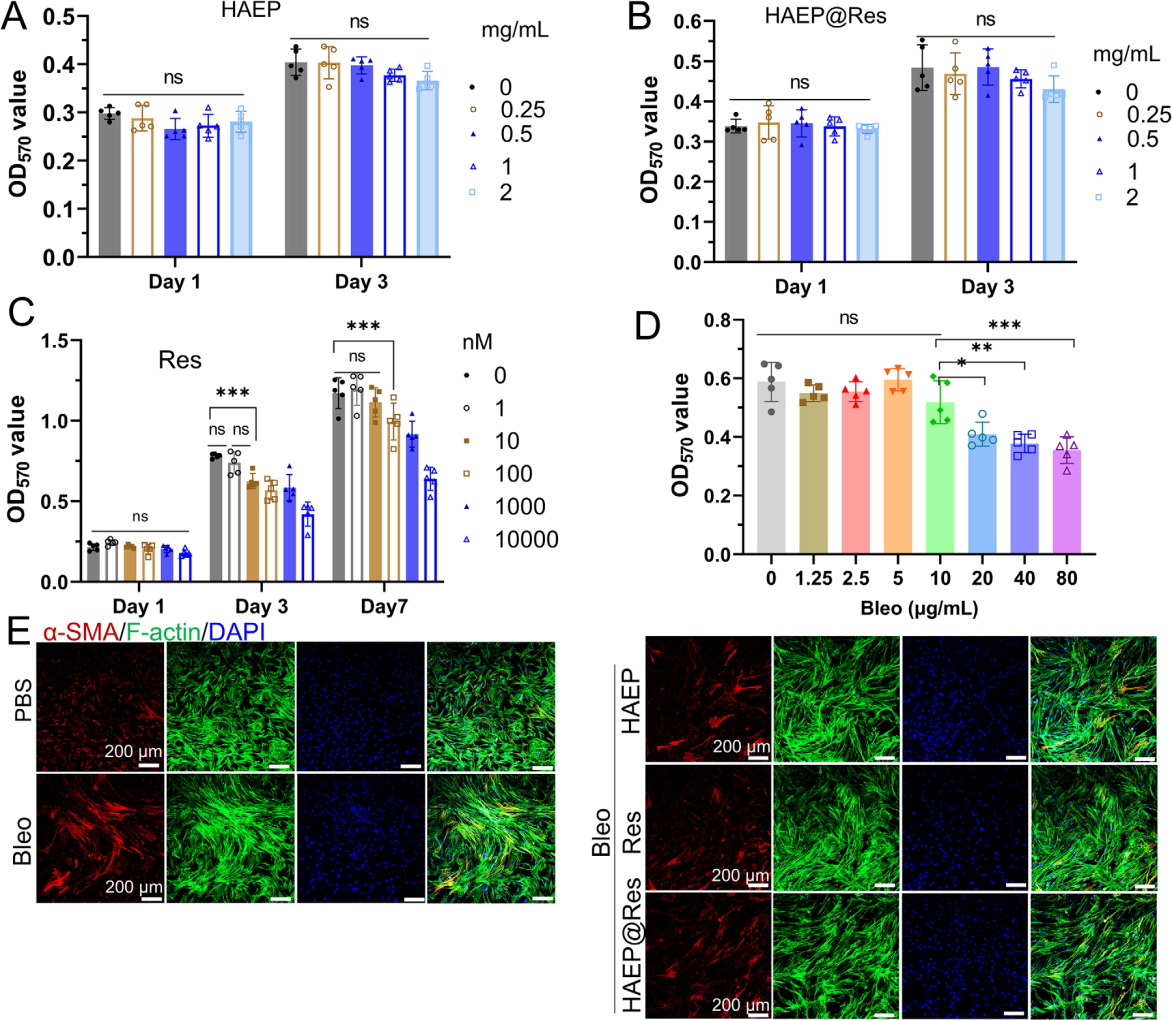
The cytotoxicity of sub‐microgels, Res, and Bleo on HLF cells in vitro: (A) HAEP alone, (B) HAEP@Res; (C) Res alone; (D) Bleo alone. (*n* = 5; *p < 0.05; **p < 0.01; ***p < 0.001; ns, not significant.) (E) IF staining of CLSM images showing the effect of sub‐microgels on expression of α‐SMA in HLFs post‐Bleo exposure. (red: α‐SMA; green: F‐actin; blue: DAPI).

To evaluate the cytotoxicity of HAEP and HAEP@res sub‐microgels in vitro, the viability of normal lung fibroblast HLF cells was assessed using the MTT assay. As shown in Figure 2A,B, although a slight increase in cytotoxicity was observed at higher concentrations, both HAEP and HAEP@Res sub‐microgels displayed no significant difference in cytotoxicity compared with the control group at concentrations range of 0–2 mg mL^−1^ on day 1 and day 3. In parallel, the in vitro cytotoxicity of free Res was assessed in HLF cells (Figure 2C). At low doses (1–100 nm), Res exhibited negligible cytotoxicity, with cell viability maintained above 80%. However, at high doses (1000–10 000 nm), Res induced significant cytotoxicity, resulting in markedly reduced cell viability compared with control group. These findings suggest that the low cytotoxicity of HAEP and HAEP@Res sub‐microgels is attributed to the inherently biocompatible nature of HAEP and its constituent polymers (HA, EGCG, and PBA). Moreover, encapsulation of Res within HAEP likely mitigates toxicity by enabling controlled drug release, thereby reducing the immediate exposure of cells to free Res.

The HLF cell viability was also used to evaluate the cytotoxicity of free Bleo (Figure 2D). Bleo at the low doses (0–10 µg mL^−1^) showed no significant cytotoxicity compared with control group, whereas the HLF cell viability was markedly reduced at the higher doses (20–80 µg mL^−1^). Based on these results, 10 µg mL^−1^ was selected for subsequent experiments. Following Bleo exposure, HLF cells were treated with HAEP, Res alone, or HAEP@Res sub‐microgels. As expected, Bleo stimulation induced upregulation of the expression of alpha smooth muscle actin (α‐SMA) in HLFs with strong immunofluorescence (IF) intensity of α‐SMA, indicating fibroblast‐to‐myofibroblast differentiation (Figure 2E). In contrast, treatment with HAEP, Res, or HAEP@Res reduced the expression of α‐SMA in HLFs, with the strongest effect observed in Res alone and HAEP@Res groups. The semi‐quantitative results also showed that the expression of α‐SMA was significantly downregulated in the HAEP@Res group compared to the Bleo group (Figure S2). Nevertheless, further studies are required to elucidate the underlying mechanisms of fibroblast‐to‐myofibroblast transition in vitro.

### 2.3 Antioxidant Activity of HAEP@Res Sub‐Microgels In Vitro

**FIGURE 3:**
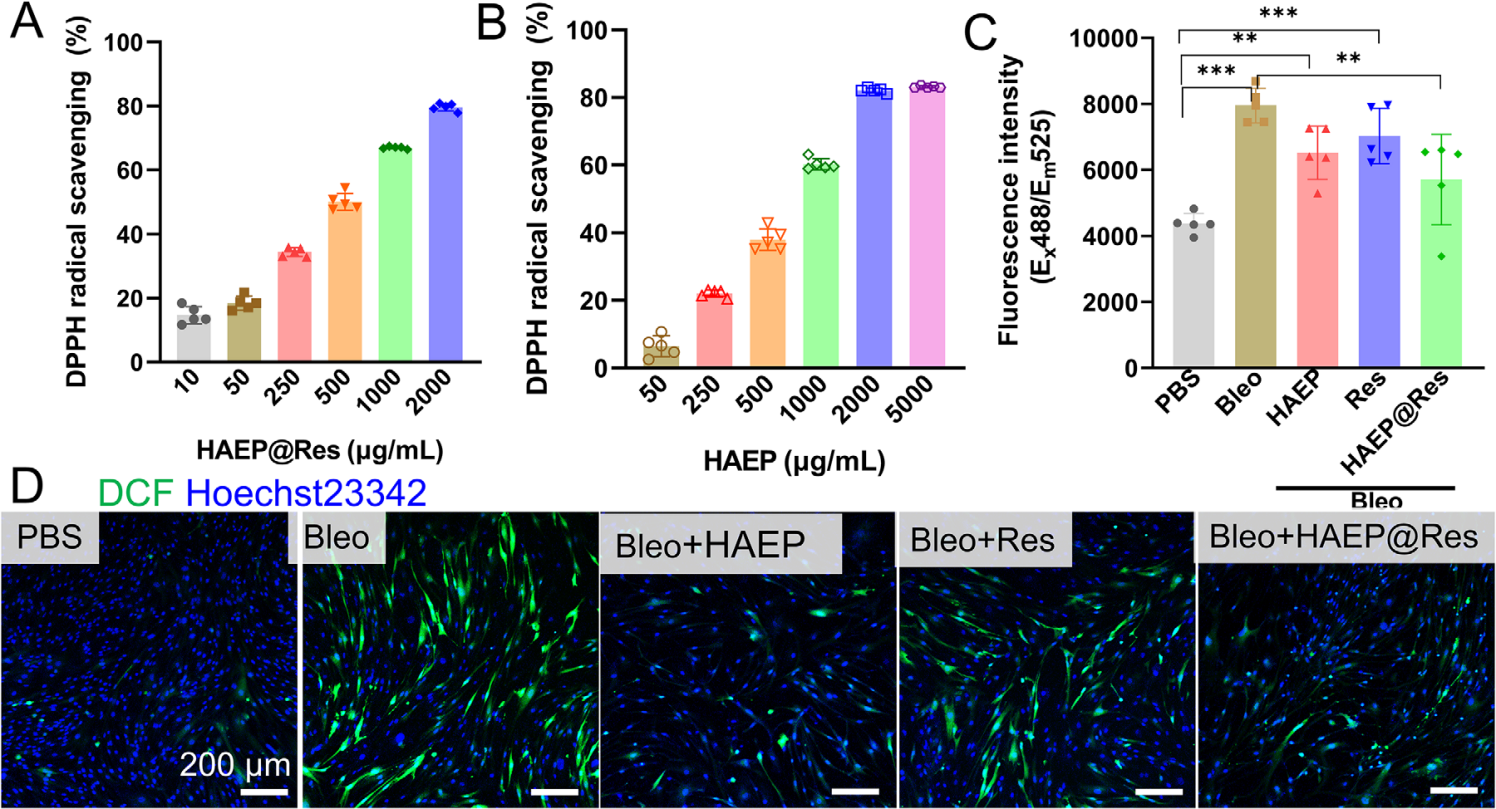
The evaluation of antioxidative properties of sub‐microgels. (A, B) Analysis of the radical scavenging levels of sub‐microgels by a DPPH assay. The stable free radical species, reactions with the radical scavenger, and the rate of radical scavenging can be measured by the colorimetric change of DPPH at 517 nm of absorbance (*n* = 5). (C) The oxidized H2DCFDA fluorescence signal intensity in HLF cells was quantified by a microplate reader (*E*_x_/*E*_m_ = 488 nm/535 nm) (*n* = 5, **p < 0.01; ***p < 0.001). (D) The alleviation of oxidative stress in L929 cells was observed via ROS probe (H2DCFDA) staining after treatments of HLF cells.

Due to the free phenol groups and the dynamic nature of boronic esters in the HAEP sub‐microgels, the sub‐microgels exhibit promising antioxidative properties [42]. Herein, we evaluated the antioxidant activity of HAEP and HAEP@Res in vitro through the 2,2‐diphenyl‐1‐picrylhydrazyl (DPPH) free radical scavenging assay and intracellular ROS assay with an oxidation‐sensitive fluorescent dye, 2’, 7’‐dichlorodihydrofluorescein diacetate (DCFHDA) (Figure 3). The DPPH assay showed a concentration‐dependent increase in free radical scavenging for both HAEP and HAEP@Res, reaching approximately 80% at 2000 µg mL^−1^ (Figure 3A,B). Based on the aforementioned ^1^H NMR calculation, 2000 µg mL^−1^ of HAEP contains around 33 µg mL^−1^ of EGCG, which aligns with the antioxidant activity of free EGCG shown in Figure S1. Over 80% scavenging was achieved at around 28 µg mL^−1^ (60 µm). These findings indicated that HAEP sub‐microgels effectively scavenged DPPH radicals, and that the anti‐oxidative effect was successfully maintained after the Res was incorporated within the composite HAEP sub‐microgels.

To further confirm intracellular ROS scavenging, HLF cells were stimulated with Bleo (10 µg mL^−1^) and treated with HAEP, Res alone, or HAEP@Res. The 2′,7′‐dichlorodihydrofluorescein diacetate (DCFHDA), a cell‐permeable non‐fluorescent probe, is hydrolyzed by intracellular esterase to DCFH and subsequently oxidized to highly fluorescent DCF by the intracellular ROS. As shown in Figure 3C, Bleo stimulation significantly increases DCF fluorescence in HLFs. Both HAEP and Res alone slightly reduced DCF fluorescence, whereas HAEP@Res markedly suppressed it. Consistently, IF images and semi‐quantitative analysis results showed a substantial reduction in DCF signal in HLFs treated with HAEP@Res (Figure 3D and Figure S3). This enhanced effect is likely due to synergistic activity: HAEP sub‐microgels scavenges ROS directly [26, 43], while Res additionally inhibits pro‐inflammatory responses in HLFs [44].

Next, we evaluated the effects of HAEP@Res microgels on the pro‐inflammatory response of RAW264.7 macrophages in vitro (Figure S4). Quantitative real‐time PCR (qPCR) analysis showed that exposure to Bleo for 24 h markedly increased the expression of pro‐inflammatory genes *Tnf*, *Il1b*, and *Il6* compared with the PBS control. Treatment with HAEP, Res, or HAEP@Res significantly reduced the expression of these genes. Notably, *Tnf* expression was significantly lower in HAEP@Res‐treated cells than in cells treated with HAEP or Res alone (Figure S4A).

### 2.4 The Biodistribution and Retention Time of HAEP@Res Sub‐Microgels In Vivo

**FIGURE 4:**
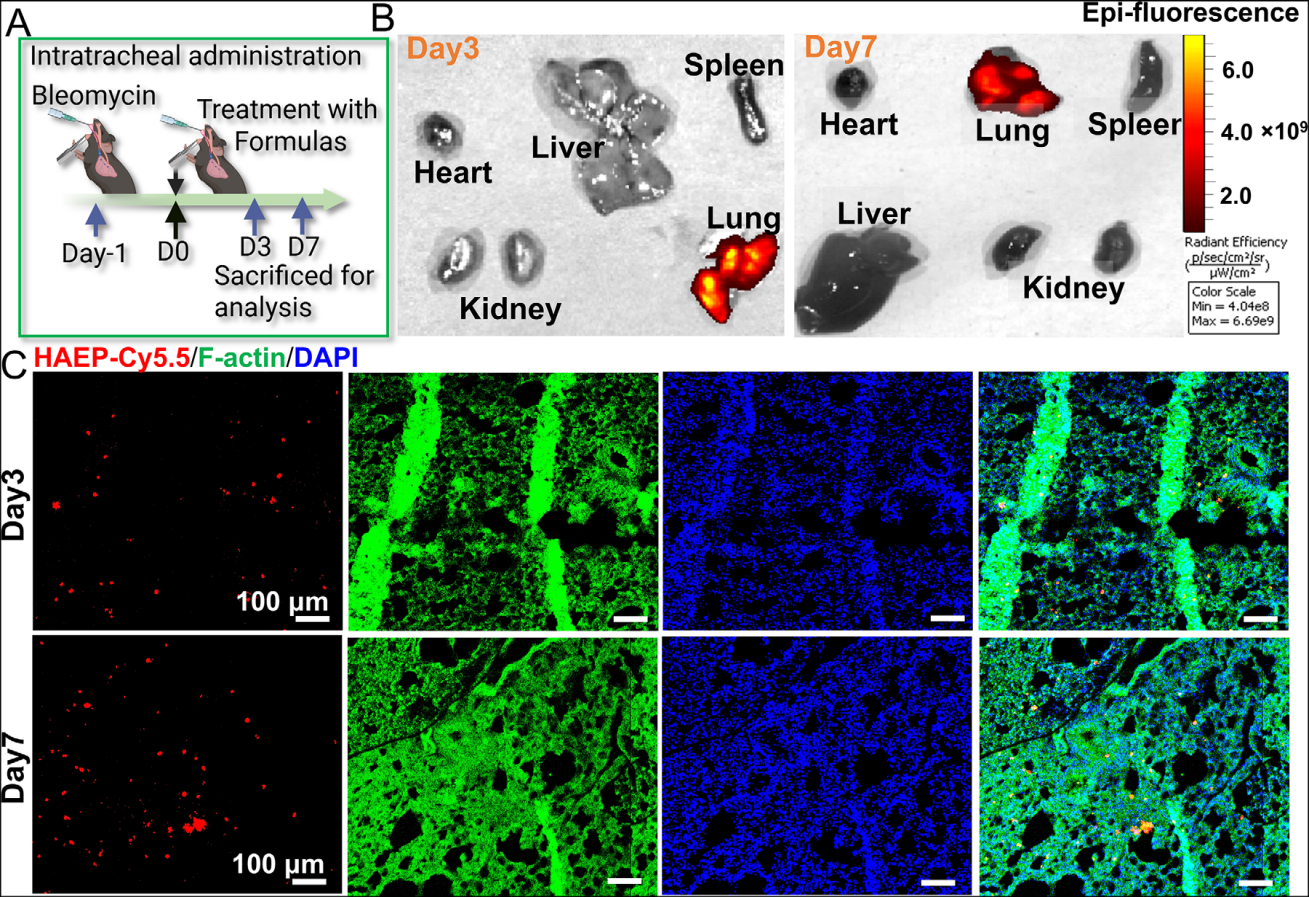
(A) Schematic illustration of the establishment and treatment schedule of Bleo‐induced ALI mouse model. (B) Biodistribution of Cy5.5‐HAEP sub‐microgels in vivo after intratracheal administration was determined by IVIS imaging in the major organs (heart, liver, spleen, lung, and kidney). (C) IF staining showing the biodistribution of sub‐microgels in the lung tissue.

Based on the aforementioned in vitro results, the therapeutic efficacy of HAEP@Res was further evaluated in a mouse Bleo‐induced ALI model, which is widely used to investigate disease progression and assess therapeutic interventions for interstitial lung injury [45]. In this study, we first investigated the in vivo biodistribution of HAEP sub‐microgels labeled with Cy5.5 (Figure 4). We employed near‐infrared fluorescence using the in vivo imaging system (IVIS) to evaluate the lung‐targeting capability of HAEP at various time points in the ALI murine model. This approach enabled longitudinal tracking of their distribution in various organs and provided insight into their therapeutic potential. We intratracheally administered Cy5.5‐HAEP sub‐microgels after Bleo challenge (Figure 4A). Strong fluorescence signals were detected in the lungs, while signals in other organs were almost absent on day 3 and day 7 (Figure 4B). To further estimate the local deposition, lung tissues from mice treated by Cy5.5‐labeld HAEP were analyzed by IF staining (Figure 4C). The results revealed strong red fluorescence signals within the lung parenchyma. This enhanced retention may be attributed to the sub‐micrometer size of the microgels, as their relatively large geometric size reduces uptake by alveolar macrophages [23]. Taken together, the above results suggest that the HAEP sub‐microgels achieve effective lung‐localized deposition and sustained retention for up to 7 d, supporting their potential for prolonged local drug delivery and release.

### 2.5 Therapeutic Efficacy of HAEP@Res Sub‐Microgels in ALI Mice

**FIGURE 5:**
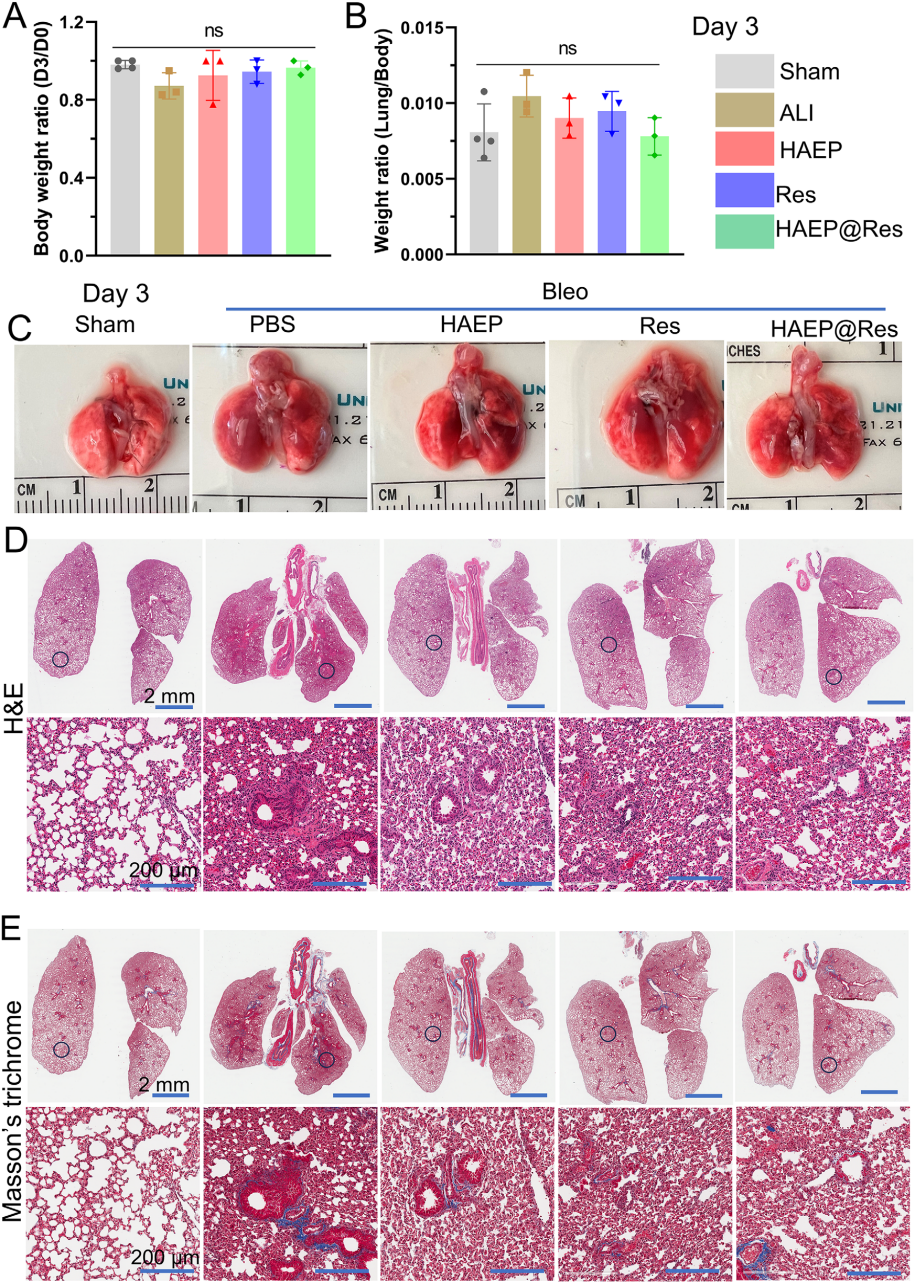
Therapeutic efficiency of HAEP@Res in the murine ALI model on day 3 post‐treatment. (A) Body weight ratio on day 0 and day 3 (*n* = 3–4). (B) Lung‐to‐body weight ratio on day 3 (*n* = 3–4). (C) Macroscopic images of lung tissues from Sham control, PBS, HAEP, Res, and HAEP@Res groups after 3 d. (D,E) Histological analysis of lung sections after 3 d of treatment, including representative H&E‐stained sections (D) and Masson's trichrome staining (muscle fibers and erythrocytes: red; collagen: blue; and nuclei: black‐purple) (E). ns, not significant.

**FIGURE 6:**
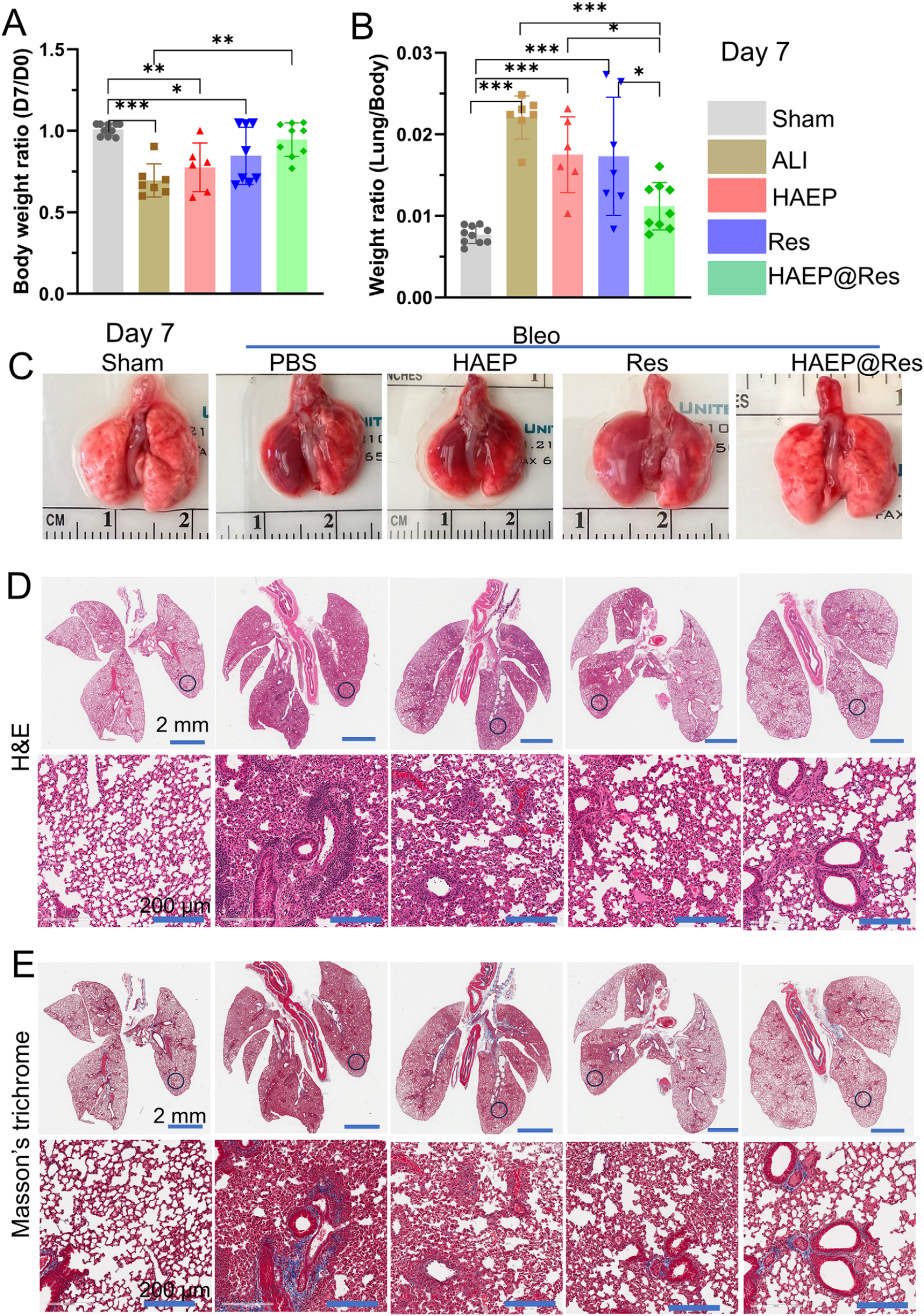
Therapeutic efficacy of HAEP@Res in the mouse ALI model on day 7 post‐treatment. (A) Body weight ratio on day 0 and day 7 (*n* = 6–8). (B) Lung‐to‐body weight ratio on day 7 post‐treatment (*n* = 6–8). (C) Macroscopic images of lung tissues from Sham control, PBS, HAEP, Res, and HAEP@Res groups after 7 d. (D, E) Histological analysis of lung sections after 7 d treatment, including representative H&E stained sections (D) and Masson's trichrome staining (muscle fibers and erythrocytes: red; collagen: blue; and nuclei: black‐purple) (E). The blue circle highlights the region magnified in the corresponding H&E and Masson's trichrome images. *p < 0.05; **p < 0.01; ***p < 0.001.

We subsequently evaluated the therapeutic efficacy of HAEP@Res on lung defense in vivo. We first evaluated two lung injury indices: body weight ratio (D3/D0 or D7/D0) and the lung‐to‐body weight ratio at day 3 and day 7, respectively (Figures 5A,B and 6A,B). At day 3, the body weight ratios (D3/D0) of Bleo‐treated mice decreased compared with the baseline Sham group (Figure 5A), suggesting weight loss following three days of injury. Although no significant difference in body weight ratio was observed between the treated groups and the Sham group after three days of treatment, lung edema was still evident. In addition, the lung‐to‐body weight ratio was higher in the Bleo group compared with the Sham and the treatment groups (Figure 5B). As expected, more pronounced therapeutic effects were observed at day 7 compared with day 3. (Figure 6A,B). The body weight ratio (D7/D0) of the HAEP@Res group was significantly improved relative to the Bleo group (Figure 6A). Moreover, the lung‐to‐body weight ratio was reduced, indicating effective mitigation of lung edema (Figure 6B).

Next, the macroscopic appearance of lungs from each group was examined (Figures 5C and 6C). Consistent with the injury index results, lungs from the Sham group appeared pink with a smooth surface, whereas those from Bleo group were dark red with pronounced congestion at both day 3 and day 7. In the treatment groups, congestion was partially alleviated, with the most notable improvement observed in the HAEP@Res group at day 3 (Figure 5C). However, the overall reversal of these congestive sites remained limited, which may be attributed to the early stage of Bleo‐ALI at day 3. In contrast, congestion and hemorrhagic sites in the HAEP@Res group were largely reversed by day 7, with lung appearance approaching that of the Sham group (Figure 6C). The enhanced improvement observed at day 7 may be attributed to the sustained release and prolonged retention of Res from the HAEP sub‐microgels.

Furthermore, we performed histological analyses of lung tissues using H&E and Masson's trichrome staining (Figures 5D,E and  6D, E). In the Bleo‐exposed group, alveolar edema, alveolar wall thickening, and microthrombus formation were significantly exacerbated at both day 3 and day 7 compared with the Sham group (Figures 5D and 6D). In contrast, these pathological features were alleviated in the HAEP, Res, and HAEP@Res groups, although they remained more severe than in the Sham group at day 3 (Figure 5D). Interestingly, these pathological changes were further alleviated by day 7, with the HAEP@Res group demonstrating the most pronounced improvement and exhibiting lung architecture closely resembling that of the Sham group (Figure 6D). In ALI, continuous damage to alveolar cells can induce the release of pro‐fibrotic factors, activating fibroblast and leading to excessive collagen production and extracellular matrix deposition, potentially progressing to pulmonary fibrosis [46, 47, 48]. In our study, Masson's trichrome staining showed that collagen fiber deposition was observed in the alveolar walls of the Bleo group, whereas smaller amount of collagen fiber deposition were observed in HAEP, Res, and HAEP@Res groups (Figures 5F and  6F). Consistently, immunofluorescence staining of α‐SMA (red) showed strong expression around bronchial and alveolar walls in the Bleo group at both day 3 and day 7, indicating fibroblast activation (Figure S2). In contrast, the HAEP@Res group displayed markedly reduced α‐SMA levels, while PBS, HAEP, and Res groups exhibited persistent α‐SMA accumulation. This reduction in α‐SMA expression correlated with the improved lung histology observed in H&E and Masson's trichrome stained lungs. Collectively, these results highlight the potent therapeutic efficacy and sustained protective effects of HAEP@Res in mitigating progression and promoting lung recovery.

### 2.6 Anti‐Inflammatory Effect of HAEP@Res in ALI Mice

**FIGURE 7:**
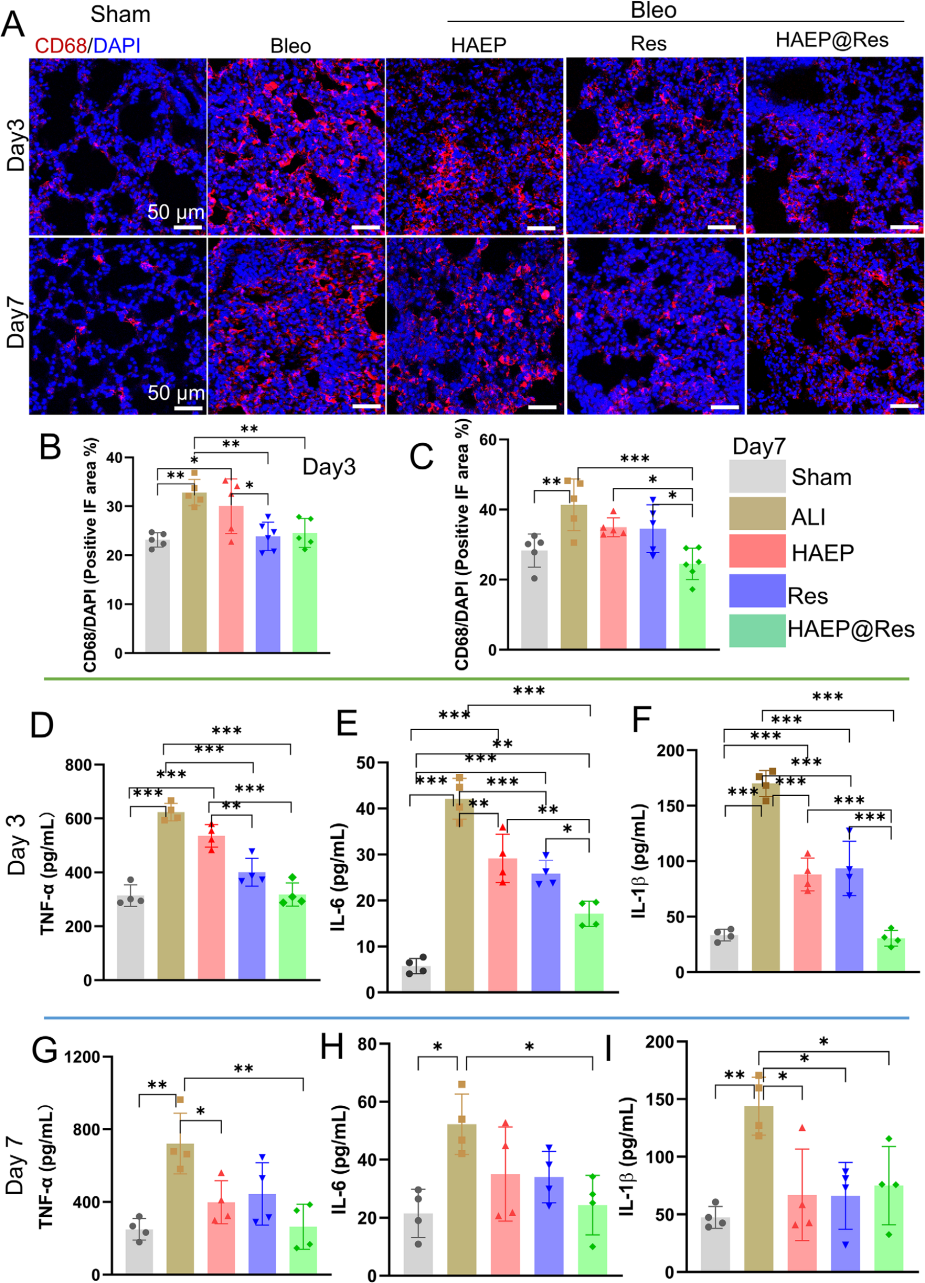
Effect of HAEP@Res on attenuation of Bleo‐induced pulmonary inflammation on day 3 and day 7 post‐treatment. (A) Representative fluorescence images visualizing CD68 positive cells in lung tissues at day 3 and day 7. Red: CD68 stained cells, blue: DAPI stained nucleus. (B, C) Semi‐quantitative data analysis CD68 positive areas in each group at day 3 and day 7, respectively. (D–I) ELISA quantification of pro‐inflammatory cytokine proteins (TNF‐α, IL‐6, and IL‐1β) in BALF at day 3(D–F), and day 7(G‐I) (*n* = 5). *p < 0.05; **p < 0.01; ***p < 0.001.

Macrophages, key immune cells in lung diseases, include two main tissue‐resident subtypes: alveolar macrophages and interstitial macrophages [49, 50, 51]. Notably, both of these macrophages can promote the progression of lung inflammation [52, 53]. To further evaluate macrophage involvement in our model, we performed CD68 IF staining to identify macrophages in the injured lung tissues (Figure 7A). As expected, lungs from the Sham group contained few CD68 positive cells, whereas the Bleo, HAEP, Res, and HAEP@Res groups exhibited markedly increased macrophage infiltration, with the most pronounced accumulation observed in the Bleo and HAEP groups at both days 3 and 7 (Figure 7B,C). Importantly, macrophage numbers were reduced in the treatment of groups, particularly in the HAEP@Res group, which showed a significant attenuation of macrophage infiltration compared with the Bleo group at day 7 (Figure 7C). These findings suggest that HAEP@Res modulates the inflammatory response by suppressing excessive macrophage accumulation.

To further explore the therapeutic effects of HAEP@Res on lung inflammation within the local immune microenvironment, bronchoalveolar lavage fluid (BALF) was collected to assess lung inflammation. The levels of tumor necrosis factor (TNF)‐α, interleukins (IL)‐6, and IL‐1β cytokines in BALF were determined by enzyme‐linked immunosorbent assay (ELISA). Following Bleo stimulation, the cytokine levels in the BALF were significantly elevated on days 3 and 7 (Figure 7D–I). After treatment with HAEP, Res alone, and HAEP@Res, the levels of these cytokines decreased, indicating that lung inflammation was improved. Notably, cytokine levels in the HAEP@Res group were significantly lower than those in the HAEP, Res groups on day 3 (Figure 7D–F). By day 7, the cytokine levels in Bleo group were further increased compared with day 3 (Figure 7G–I). Cytokine levels in the treatment groups were increased, but remained lower than those in the Bleo group.

**FIGURE 8:**
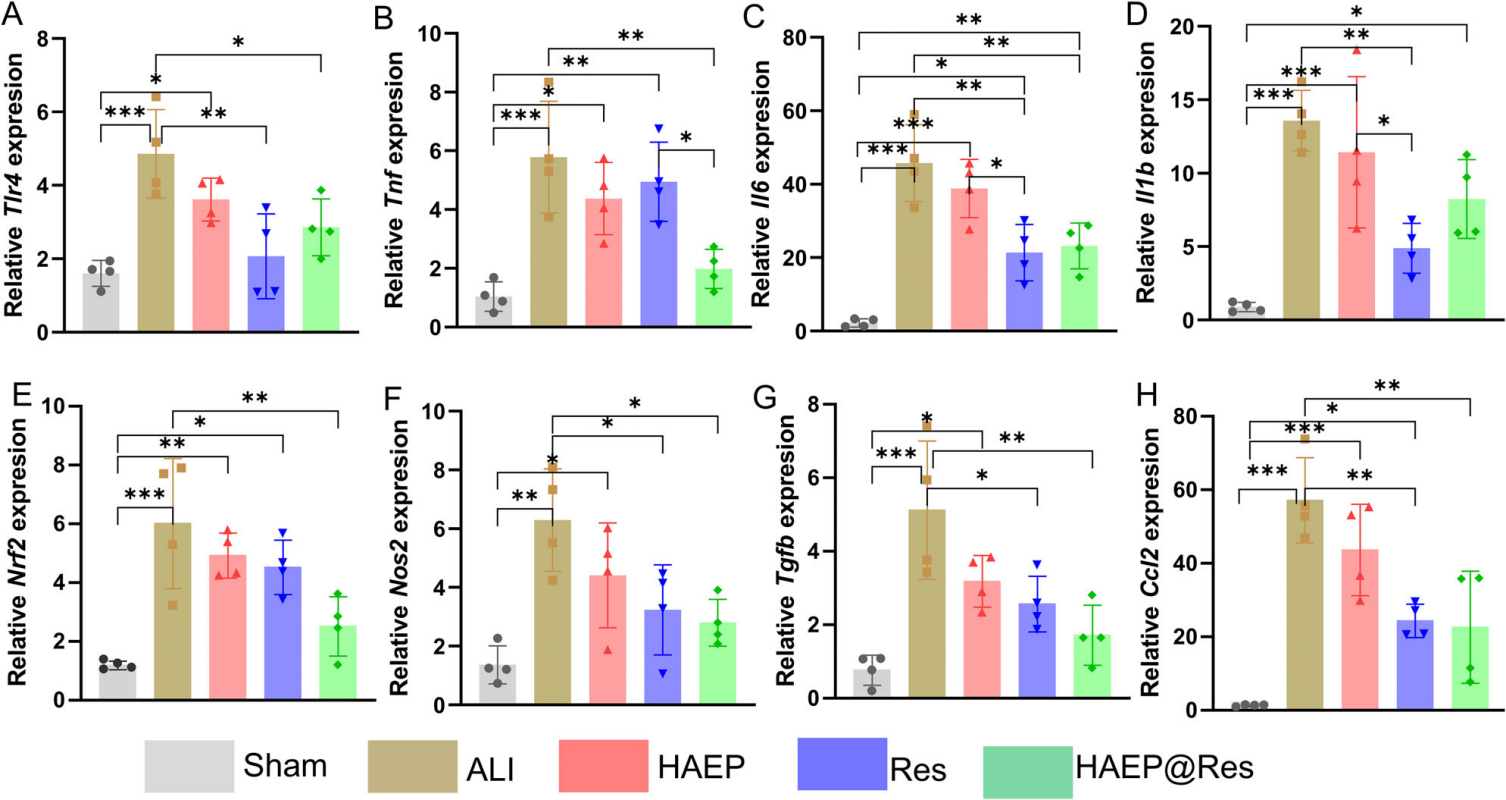
Effect of HAEP@Res on the regulation of pro‐inflammatory gene expression in lung tissues on day 7. QPCR analysis of pro‐inflammatory gene expression in lung tissues of different treatment groups: A: *Tlr4*; B: *Tnf*; C: *Il6*; D: *Il1b*; E: *Nrf2*; F: *Nos2*; G: *Tgfb*; H: *Ccl2*; *n* = 4, *p < 0.05; **p < 0.01; ***p < 0.001.

We then examined whether HAEP@Res could modulate the expression of pro‐inflammatory genes (Figure 8). The expression of *Tlr4* was significantly downregulated in the Res and HAEP@Res groups compared with the Bleo group (Figure 8A), consistent with previous studies showing that Res acts as a specific inhibitor of TLR4 signaling, thereby suppressing activation of inflammatory pathways [54]. In parallel, the expression levels of *Tnf*, *Il6*, *Il1b*, *Nrf2*, *Nos2*, *Tgfb*, and *Ccl2* were markedly elevated in the lungs at day 7 after Bleo exposure compared with those in the Sham group (Figure 8B–H). Notably, HAEP@Res treatment significantly reduced the expression of most of these proinflammatory genes compared to the Bleo group, including *Tlr4*, *Tnf*, *Il6*, *Nrf2*, *Nos2*, *Tgfb*, and *Ccl2*, while *Il1b* remained unchanged. In contrast, in the Res group, the expression levels of *Tlr4*, *Il6*, *Il1b*, *Tgfb*, and *Ccl2* were significantly decreased, whereas *Tnf* and *Nrf2* showed no significant change. Interestingly, although the levels of *Tlr4*, *Il6*, *Il1b*, *Nrf2*, *Nos2*, *Tgfb*, and *Ccl2* in HAEP@Res group were comparable to those in Res group, *Tnf* expression in HAEP@Res was significantly lower, highlighting an enhanced anti‐inflammatory effect of HAEP@Res. The possible reason that the HAEP@Res produced stronger and more consistent anti‐inflammatory effects observed with HAEP@Res compare to free Res may be attributed to its intratracheal delivery via HAEP sub‐microgel, which likely increases local drug concentration and prolongs retention time within lung tissue [23]. In contrast, free Res is more susceptible to rapid clearance, thereby reducing its therapeutic availability at the site of injury [13, 55]. Taken together, ELISA and qPCR results indicated that HAEP@Res suppresses the pro‐inflammatory response in ALI mouse by downregulating both pro‐inflammatory cytokine secretion and gene expression.

## 3 Discussion

Effective drug delivery to damaged lung tissues and the regulation of inflammation are major challenges in the clinical treatment of ALI/ARDS [1]. Microgels are lightly cross‐linked gel particles that can drastically change their volume in response to environmental factors such as redox conditions, temperature, and pH, enabling them to encapsulate and release payloads in a responsive manner [56]. In addition, microgels combine properties of hydrogels and micro‐scaled particulates [41]. In this study, HAEP sub‐microgels were formed through multiple intermolecular interactions, including esterification, dynamic boronic bonds, and hydrogen bonding, hydrophobic interactions, and π‐π stacking between HA, EGCG and PBA molecules (Scheme 1A and Figure 1A,B) [40]. When exposed to aqueous buffer, the HAEP sub‐microgels swelled, and displayed hydrodynamic diameters compared with their dry state (Figure 1C–H). The final swollen particle sizes of HAEP may reduce rapid phagocytic clearance while still enabling pulmonary deposition, consistent with previous reports that particle size between 1–5 µm are suitable for pulmonary delivery [23, 57, 58]. Moreover, HA has been widely investigated as a carrier for selective drug delivery to the inflammatory lesions via the HA‐receptor‐mediated recognition by pro‐inflammatory macrophages, thereby increasing drug accumulation at target sites and improving therapeutic index [59, 60]. Consistent with prior findings, our HA‐based HAEP sub‐microgels with biocompatibility and anti‐ROS properties exhibited at least 7 days of retention in the lung tissue (Figures 2, 3, 4), supporting sustained drug release enabled by the boronic ester dynamic covalent bonds between EGCG and PBA [43].

TLR4, a pivotal member of the Toll‐like receptor family, has been extensively studied for its role in mediating infection‐induced inflammation in past decades [28, 29, 61]. It regulates macrophage function by activating downstream inflammatory signaling pathway [32, 62]. Imai et al. demonstrated that TLR4 signaling regulates the severity of ALI, as its expression was elevated in alveolar macrophages during virus‐induced ALI, whereas TLR4 mutant mice showed natural resistance [31]. Fu et al. showed that TLR4 deficiency markedly alters the cellular and transcriptional landscape of lung cells in sepsis‐induced ALI, with macrophage TLR4 driving inflammatory signals that activate pulmonary and lymphatic endothelial cells [32]. Given these reports, Res, a TLR4‐specific signaling inhibitor, has shown therapeutic potential in various inflammatory diseases [32, 34, 44]. Therefore, HAEP@Res sub‐microgels were developed for the treatment of ALI in a murine model (Scheme 1B). Our results demonstrated that HAEP@Res sub‐microgels not only retained antioxidant properties but also further downregulated α‐SMA expression in HLFs compared with HAEP alone in vitro (Figures 2E and  3), as well as in lung tissues in vivo (Figure S5). Previous literatures showed that α‐SMA is a well‐recognized biomarker of myofibroblast activation and fibroblast‐to‐myofibroblast transition [63, 64, 65]. Myofibroblast activation is a key contributor to ALI progression following epithelial and endothelial injury [66]. Activated myofibroblasts produce excessive extracellular matrix, leading to alveolar wall thickening, reduced lung compliance, and impaired gas exchange [67]. Dysregulated or persistent activation can delay injury resolution and promote pathological lung remodeling [68]. Collectively, these findings suggest that HAEP@Res may contribute to suppressing myofibroblast activation and mitigating tissue remodeling in Bleo‐induced ALI by downregulating α‐SMA expression. However, additional studies are required to provide direct evidence for this mechanism both in vitro and in vivo.

Intratracheal administration offers the advantage of localized delivery, limiting systemic distribution to other organs [69]. However, it also faces multiple physiological barriers, including the mucus layer and pulmonary surfactant, which can capture and clear foreign particles via physical interactions [70, 71]. In this study, HAEP sub‐microgels were administered as a buffer solution via intratracheal instillation, allowing direct deposition in the lungs while minimizing systemic exposure. The freeze‐dried size of HAEP sub‐microgels ranges from 0.2 to 1.4 µm (Figure 1C–H), which is suitable for deposition in the bronchiolar and alveolar regions [23]. Under ALI conditions, alveolar macrophages and other immune cells can recognize and internalize these sub‐microgels, enhancing transient retention and cellular uptake [72]. Additionally, HA microgels have been reported to disrupt endosomes via membrane fusion, with protonation of glucuronic acid units enhancing amphiphilicity and promoting endosomal membrane lysis in the acidic environment [25]. Collectively, these size‐dependent deposition properties, immune interactions, and endosomal escape mechanisms likely contribute to the observed lung‐selective distribution and support the potential of HAEP sub‐microgels for targeted pulmonary drug delivery.

Following intratracheal injection of Bleo, the initial acute injury and inflammatory phase (days 1–7) is characterized by severe epithelial damage, infiltration of inflammatory cells, activation and release of numerous inflammatory mediators, vascular leakage, and upregulation of pro‐inflammatory cytokines and chemokines [8, 45]. To assess therapeutic potential of HAEP@Res, mice were intratracheally administered HAEP@Res and evaluated on day 7 post‐ Bleo treatment. Our results showed that the lung/body weight ratio was dramatically increased after Bleo stimulation compared with the Sham group at both day 3 and day7 (Figures 5A and  6A). The increase is consistent with elevated capillary permeability and pulmonary edema in ALI, leading to lung weight gain and the impaired gas exchange [73]. In contrast, the lung/body weight ratio was dramatically reduced in the HAEP@Res group, suggesting attenuation of pulmonary edema at day 7 (Figure 6A). Histologically, the acute phase (first week) is marked by infiltration of immune cells and red blood cells into the alveoli, and acute alveolar hemorrhage [8]. In our study, the histological analysis confirmed that alveolar edema, alveolar wall thickening, and microthrombus formation were significantly exacerbated at both days 3 and day 7 compared with the Sham group (Figures 5C–E and  6C–E). Notably, these pathological changes were alleviated in the HAEP@Res group, which showed reduced congestion and diminished infiltration of inflammatory cells (Figure 6D,E).

In order to evaluate the effect of TLR4 inhibition of HAEP@Res in a Bleo ‐induced ALI mouse model, we assessed the pro‐inflammatory cytokine secretion and gene expression in lung tissues. During lung injury, protein‐rich fluids leak and immune cells penetrate due to the disruption of the alveolar‐capillary barrier [74, 75]. Consistently, we observed that increased CD68 positive immune cells increased in the Bleo group at both day 3 and day 7 compared with Sham group (Figure 7A–C). The levels of TNF‐α, IL‐6 and IL‐1β were markedly elevated at both day 3 and day 7 following Bleo stimulation in vivo (Figure 7D–I). However, intratracheal administration of HAEP@Res reduced the levels of these inflammatory factors. Previous studies demonstrated that administration of a TLR4 inhibitor decreased the expression of TLR4, and subsequently lowered the inflammatory cytokine levels, similar to using TLR4 siRNA [76]. Our results suggested that HAEP@Res produced a stronger anti‐inflammatory effect than HAEP or Res alone. This might be due to the synergistic effect, with HAEP providing antioxidant properties and Res acting as a small‐molecule selective inhibitor of TLR4 signaling to suppress the increase in cytokine levels [43, 77, 78]. Consistent with our ELISA results, our qPCR analysis also revealed that the pro‐inflammatory gene expression was significantly downregulated in the HAEP@Res group (Figure 8), likely through the downregulation of the TLR4 signaling pathway [78]. Nevertheless, the specific mechanisms require further investigation. Together, our results suggest that HAEP@Res can prevent inflammatory response and ameliorate lung injury in a Bleo‐induced ALI mouse model.

Although promising, our current study has several limitations. First, the dose and timing of HAEP@Res administration may affect the outcome. We used a single intratracheal dose 24 h after Bleo exposure, but alternative doses, routes, or schedules may have different effects and require further optimization. Second, the mechanism by which HAEP@Res modulates TLR4 signaling pathway in ALI remains incompletely explored. We only measured a limited set of pro‐inflammatory genes and cytokines, whereas additional components and regulators of TLR4 signaling may also contribute. More comprehensive protein level and functional studies are needed to clarify these mechanisms. Finally, lung function data were not obtained, which would provide more clinically relevant insights than histological or molecular analyses alone. Therefore, our future study will focus on evaluating lung function recovery in mice following HAEP@Res treatment, including respiratory parameters and arterial blood gas measurements [79, 80].

## 4 Conclusion

In this study, we developed novel HAEP@Res sub‐microgels that combine intrinsic antioxidant activity with lung‐targeting capacity for ALI therapy in a mouse Bleo ‐induced ALI model. The HAEP@Res sub‐microgels exhibited excellent biocompatibility and effectively suppressed intracellular ROS in vitro. Furthermore, the HAEP@Res intratracheal administration markedly alleviated histological lung injury, and significantly downregulated pro‐inflammatory cytokine secretion and gene expression in vivo. Collectively, these findings demonstrate that HAEP@Res sub‐microgels represent a biocompatible and versatile drug delivery platform, offering a promising therapeutic strategy for ALI/ARDS and other pulmonary diseases.

## 5 Experimental Section

### 5.1 Synthesis of HA‐PBA‐EGCG Loading Res (HAEP@Res) Sub‐Microgels

The HAEP@Res sub‐microgel was prepared via an in situ conjugation reaction among HA, PBA, and EGCG, as described in our previous reports [81, 82]. Briefly, HA (50 mg; 7.5 kDa, Bloomage Biotech) was dissolved in 10 mL of milliQ water. The pH of this solution was subsequently adjusted to 6.5 by adding MES buffer (1 m, pH 5.5; Fisher Chemical). Then, EGCG (2.52 mg; AstaTech) and 3‐aminophenylboronic acid (PBA; 24.84 mg; AstaTech) were dissolved in 0.5 mL DMSO. Res drug (5 mg; TAK‐242, HY‐11109m MCE) was directly added into EGCG/PBA mixture solution, which was then dropped into the HA aqueous solution and left at room temperature (RT) for 20 min. Next, 4‐(4,6‐dimethoxy‐1,3,5‐triazin‐2‐yl)‐4‐methylmorpholinium chloride (DMTMM; 772.86 mg; TCI Chemical) were added dropwise, followed by a 3‐day reaction at RT. The final mixtures were dialyzed (molecular weight cutoff of 3.5 kDa, MWCO, Spectrum) at 4°C for 3 days, freeze‐dried, and prepared for use. As a negative control, HAEP sub‐microgels were prepared using a similar procedure. For the preparation of fluorescein‐labeled HAEP sub‐microgels, cyanine 5.5 (Cy5.5)‐labeled HA was first synthesized via an amination reaction between fluorescein cyanine 5.5 amine (Lumiprobe, 0.1% w/w relative to HA) and HA. Subsequently, the Cy5.5‐labeled HAEP sub‐microgels were prepared following the previously described procedure [81, 82].

### 5.2 Characterization of HAEP and HAEP@Res Sub‐Microgels

The morphology of the microparticles was visually characterized by a scanning electron microscope (SEM, FEI Quanta 200). The size of the sub‐microgel particles was calculated by ImageJ software. The particle size distribution graph was analyzed by Originlab software. The hydrodynamic diameter, and polydisperse index (PDI) were measured via dynamic light scattering (DLS) using a Zetasizer Nano ZS (Malvern).

### 5.3 HPLC Analysis

The drug content and encapsulation efficiency of HAEP@Re sub‐microgels were determined using HPLC analysis method. Briefly, the microgels (1 mg mL^−1^) were incubated with 1 mL DPBS at 37°C for various time points (3 h, 6 h, 1, 3, 7, 14, 21, and 28 d). At each time point, the supernatant DPBS solution (1 mL) was collected after centrifugation at 5,000 rpm for 5 min at 4°C. After, an equal amount of fresh DPBS was added to resuspend the sub‐microgels sample. The collected supernatant samples were analyzed using an Agilent Poroshell EC‐C18 column 2.7 um (4.6×100 mm) at 10°C. Mobile phase conditions were acetonitrile with trifluoroacetic acid (0.1% v/v) in HPLC‐grade water with 10:90 (v/v) at a flow rate of 1.0 mL min^−1^. Drug was detected using an Agilent 1260 with a diode array and a refractive index detector (254 nm). Drug content was determined relative to peak areas of drug standards (6.25–100 µg mL^−1^). Each sample was tested in quadruplicate. The standard curve of Res drug was set up as shown in Figure S6. The drug LE and EE of Res were calculated based on the standard curves, according to the following formula:LE(wt%) = weight of loaded drug/total weight of loaded drug and polymer × 100

$$\mathrm{EE}\left(\mathrm{wt}\%\right)=\mathrm{weight}\;\mathrm{of}\;\mathrm{loaded}\;\mathrm{drug}/\mathrm{weight}\;\mathrm{of}\;\mathrm{drug}\;\mathrm{in}\;\mathrm{feed}\times 100$$

### 5.4 Cell Culture

HLF cells were isolated from deidentified normal lungs obtained from the Nebraska Organ Retrieval System in accordance with the guidelines of the University of Nebraska Institutional Review Board [83]. The HLF were cultured at 37°C incubator with 5% CO_2_ in DMEM/F12 (Gibco) containing 10% (v/v) fetal bovine serum (FBS, Gibco) and 1% (v/v) penicillin‐streptomycin (P/S, Gibco). All the experiments were performed using cells passage 6 or lower.

### 5.5 In Vitro Cytocompatibility of HAEP and HAEP @Res Sub‐Microgels

The cytotoxicity of HAEP and HAEP@Res sub‐microgels on HLFs cells were evaluated by MTT assays (3‐(4,5‐dimethylthiazol‐2‐yl)‐2,5‐diphenyl‐2H‐tetrazolium bromide, Sigma). HAEP and HAEP@Res sub‐microgels were first resuspended in the culture medium at various concentrations (0, 0.25, 0.5, 1, 2 mg mL^−1^) and then incubated at 37°C for 24 h. The extracts media were collected after the HAEP or HAEP@Res sub‐microgel mixture were centrifugated at the speed of 4500 rpm for 30 min at 4°C. The HLFs were seed into 48‐well plate (1×10^4^ per well) and cultured in the DMEM/F12 media. After incubation overnight at 37°C, the culture media were removed, and the cells were cultured in extract media for further 24 h. Then, the cells were washed with PBS after the extract media were discarded. About 450 µL of culture medium containing 50 µL of MTT solution (5 mg mL^−1^ in PBS) was added to each well, followed by incubation for 4 h. Subsequently, the medium was discarded, and 500 µL of dimethyl sulfoxide (DMSO) was added and shaken at 100 rpm for 30 min to dissolve the blue crystals in each well of the 48‐well plate. Finally, the solution was transferred from the 48‐well plate into a 96‐well plate at 50 µL per well and the OD value was measured at 570 nm (OD_570_) using a microplate reader (BioTek Synergy H1 model). The untreated cells incubated with the normal culture media served as a control. The cell toxicity of Res alone and Bleo (BML‐AP302, Enzo) alone at various concentrations was also evaluated using similar procedure aforementioned. Five independent replicates were performed for each sample.

### 5.6 Immunofluorescence Staining of α‐SMA on HLF

To observe the effects of HAEP, Res, and HAEP@Res on fibrogenic activity of HLF cells post‐ Bleo treatment in vitro, alpha‐smooth muscle actin (α‐SMA) antibody is used as a marker. Briefly, after HLFs cells (1×10^5^ per well in 24‐well plates) were cultured in cell culture medium at 37°C overnight, HLFs were further cultured in media with/without Bleo (10 µg mL^−1^) for another 24 h. Meanwhile, the extracts media were collected after the HAEP or HAEP@Res sub‐microgel solution (2 mg mL^−1^) were centrifugated at the speed of 4500 rpm for 30 min at 4°C. Then, the cells were treated with collected sub‐microgel extract medium containing Bleo (10 µg mL^−1^) for further 24 h. The cultured HLF cells were washed three times in PBS and fixed in 4% paraformaldehyde (PFA) at 4°C for 4 h. Then, the samples were washed three times in PBS and permeabilized by using 0.2% Triton X‐100 (Sigma) for 10 min at room temperature (RT). Subsequently, the samples were blocked in 1% BSA at 4°C overnight after three PBS washes. The Fixed cell samples were incubated with α‐SMA‐Cy3 (C6918, Sigma) and F‐actin phalloidin conjugates (ActinGrenn 488, Invitrogen) at RT for 2 h and further incubated with DAPI (1: 1000, Invitrogen, D1306) for 30 min at RT. Finally, the stained samples were washed one time in PBS and imaged with a confocal laser scanning microscopy (CLSM, Zeiss, LSM 800).

### 5.7 DPPH Free Radical Scavenging Activity

The radical scavenging activity of HAEP and HAEP@Res sub‐microgels was measured by observing colorimetric changes of DPPH (Alfa Aesar) according to the previous report [81]. Briefly, the sub‐microgels aqueous solution (200 µL) at various concentrations (10, 50, 250, 500, 1000, 2000 µg mL^−1^) were added into DPPH ethanol solution (0.1 mM, 400 µL). The mixture was incubated under shaking in a dark place at 37°C for 30 min. Next, the absorption (A) of the samples was tested at 517 nm using a UV−vis instrument. The DPPH free radical scavenging efficiency was calculated by the following formula: DPPH radical scavenging % = (*A*_0_ − *A*_h_)/*A*_0_ × 100, where *A*_0_ and *A*_h_ are the absorptions of the blank (DPPH + ethanol) and the absorption of the sub‐microgel samples (DPPH + sub‐microgels), respectively. The radical scavenging activity of EGCG at various concentrations was also evaluated using similar method. Five independent replicates were performed for each sample.

### 5.8 In Vitro Measurement of Intracellular ROS

In this study, intracellular ROS levels were measured using the fluorescent probe, H2DCFDA (EMD Millipore), which is hydrolyzed to H2DCF and subsequently oxidized to the fluorescent product DCF after crossing the plasma membrane. The sub‐microgel extract medium was pre‐prepared after sub‐microgels were co‐incubated in the cell culture medium (2 mg mL^−1^) overnight at 37°C. After HLFs cells (1×10^4^ per well in 48‐well plates) were cultured in cell culture medium at 37°C overnight, HLFs were further cultured in media with/without Bleo (10 µg mL^−1^) for another 24 h. The pure culture media were used as control. Then, the cells were treated with collected sub‐microgel extract medium containing Bleo (10 µg mL^−1^) for overnight. The experimental groups were established as follows: cells pretreated with PBS, Bleo alone, Bleo with HAEP sub‐microgels, and Bleo with HAEP@Res sub‐microgels. After being washed with DPBS buffer three times, the cells were incubated with culture medium containing H2DCFDA (10 µm) and further incubated for another 45 min in the dark at 37°C. Subsequently, the cells were washed with DPBS buffer three times, and the intracellular ROS concentration was measured by detecting the DCF fluorescence intensity with excitation at 488 nm and emission at 525 nm through a microplate reader. The cells (1×10^5^ per well in 24‐well plates) were also imaged using a CLSM to visualize the DCF fluorescence after treated by the same procedure. Five independent replicates were performed for each sample.

### 5.9 Bleo‐Induced Acute Lung Injury Model and In Vivo Treatment with HAEP, Res, or HAEP@Res

The mouse ALI model was established through intratracheal injection of Bleo, which has been widely accepted as a classical animal model of pulmonary injury [45]. All animal procedures were approved by the Institutional Animal Care and Use Committee (IACUC) at University of Nebraska Medical Center. In brief, C57BL/6 mice (male, 6‐week‐old) purchased from Charles River Laboratories were housed in an animal feeding system of individually ventilated cages under standard laboratory housing conditions (55±5% humidity, 22±2°C, 12 h light‐dark cycle, respectively) for one week. Afterward, mice were randomly divided into 5 groups: sham mice (1 group) and Bleo ‐exposed mice (four groups). All mice were first anesthesia by peritoneal administration of ketamine/xylazine mixture solution (30 g/0.4 mL). In four groups, intratracheal Bleo (2.5 mg kg^−1^, ≈50 µL) was instilled intratracheally to the lungs under anesthesia to induce acute lung injury. Sham control mice underwent intratracheal instillation of saline. The ALI mice were followed by administration of PBS (injury only control, *n* = 12), HAEP (50 mg/kg, 50 µL, *n* = 12), Res (3 mg kg^−1^, 50 µL, *n* = 12), HAEP@Res (50 mg kg^−1^, 50 µL, *n* = 12). Three mice from each group were euthanized on day 3 post‐treatment, and the remaining mice were euthanized on day 7 post‐treatment. Lung tissues and broncho‐alveolar lavage fluid (BALF) were collected for further experiments.

### 5.10 IVIS Imaging

The retention time of intratracheally injected HAEP sub‐microgels in the lung was evaluated using IVIS (IVIS Spectrum, PerkinElmer), which detected fluorescence from Cy5.5 labeled HAEP sub‐microgels. Briefly, Cy5.5‐labeled HAEP sub‐microgels (50 mg kg^−1^, 50 µL) were resuspended in DI water and then intratracheally injected into lung following ALI model procedure. After 3 and 7 d, the rats were sacrificed in a CO_2_ chamber and immediately dissected to remove the lung, heart, liver, spleen, and kidney. The organs were quickly washed with PBS buffer to remove excess blood from the necropsy process. The extracted organs were placed on a black, non‐emitting background for fluorescence analysis. The excitation filter was set at 644 nm, and the emission filter was set at 665 nm.

### 5.11 Lung Edema Measurement

The extent of pulmonary edema was evaluated using the lung index, Mice were weighed before euthanasia, the entire lung tissue was isolated from the thoracic cavity of each mouse and weighed. The lung indices were determined by calculating the ratio of the lung weight to the total body weight. The body weight indices were determined by calculating the ratio of the total body on day 0 (D0) to the total body weight on day 3 (D3) or day 7 (D7).

### 5.12 Histological Analysis of Lung Injury

H&E and Masson's trichrome staining were used to measure the lung injury. Fresh lung tissues were harvested and fixed with 4% paraformaldehyde for about 24 h and then embedded in paraffin and sectionalized. Each section was 5 µm thick and was stained with hematoxylin eosin (H&E) and Masson's trichrome following the standard protocol.

### 5.13 IF Analysis of Local Inflammation

The isolated lung tissues were dissected and then fixed in 4% PFA solution and dehydrated in 30% sucrose. Then, the specimen was embedded in optimal cutting temperature (OCT) compound within a cryo‐mold and cut into 12 µm‐thick sections at −20°C using a cryotome (CM1850, Leica). After being immerged in methanol for 5 min, all sections were permeabilized and blocked with 0.1% Triton X‐100 and 5% goat serum in PBS for 2 h, followed by incubation with primary antibody CD68 (AbboMax, 500–6074, 1:100) overnight at 4°C. After sequential incubation with the secondary antibody, Alexa Fluor TM 568 goat anti‐mouse IgG (H + L) (1:400, Thermo Fisher Scientific) for 2 h and DAPI (Invitrogen, D1306, 1:1000) for 30 min at room temperature, the stained samples were imaged using a confocal laser scanning microscopy (CLSM, Zeiss LSM800). The inflammatory effect was semiquantitatively evaluated by calculating the ratio of CD68 positive areas to DAPI positive area, which were measured using ImageJ software.

### 5.14 Bronchoalveolar Lavage Fluid (BALF) Extraction and Enzyme‐Linked Immunosorbent Assay (ELISA) Assay

The mice were sacrificed at the indicated time point, and their trachea was exposed immediately and intubated with a polyethylene catheter. Then, the lungs were lavaged with three separate volumes of 0.5 mL PBS, supplemented with EDTA. The collected BALF was centrifuged at 4°C and1000 rpm for 10 min, and the supernatant was collected. Cytokine levels of TNF‐α, IL‐6, and IL‐1β in BALF were measured using mouse‐specific ELISA kits (R&D Systems) according to the manufacturer's instructions.

### 5.15 Isolation and Quantification of RNA via qPCR Analysis

The total RNA was extracted from mouse lung tissues by subsequent treatments with TRIzol Reagent (Ivitrogen), ethanol, and centrifugation. The extracted total RNA was then purified by using PureLink RNA Mini Kit (Invitrogen). The purity of RNA was determined by ultraviolet spectrophotometer (NanoDrop One, Thermo Fisher Scientific). Subsequently, Complementary DNA (cDNA) was synthesized by following the protocol of an iScript cDNA synthesis kit (Bio‐Rad Laboratories). For the quantitative analysis of the total RNA expression, qPCR amplification was performed in a StepOnePlus Real‐Time PCR System (Thermo Scientific) with the use of SYBR Green Supermix (Bio‐Rad). The cDNA samples were analyzed for genes of interest and for the housekeeping gene *Gapdh*. The target genes included *Tlr4*, *Tnf*, *Il6*, *Il1b*, *Nrf2*, *Nos2*, *Tgfb1*, and *Ccl2*, as listed in Table S1. The relative transcription level of each target gene was calculated using the comparative Ct (2^−ΔΔCt^) method.

### 5.16 Statistical Analysis

All quantitative data were expressed as mean ± standard deviation (Mean ± SD). Statistical analysis was performed using an unpaired Student's t‐test for comparisons between two groups, while comparisons among multiple groups were conducted using one‐way and two‐way analysis of variance (ANOVA) with a Tukey's multiple comparison test. All analyses were performed using GraphPad Prism software. A *p*‐value <0.05 was considered significant for all statistical analyses.

## Funding

Scientist Development Award (B.L.), University of Nebraska Collaboration Initiative Grant (B.L.), NIH (R21 HL170127‐01) (H.J.W., B.D.), NIH (1R01HL152160‐01) (H.J.W)

## Conflicts of Interest

The authors declare no conflicts of interest.

## Supporting information




**Supporting File**: adhm71077‐sup‐0001‐SuppMat.docx.

## Data Availability

The data that support the findings of this study are available from the corresponding author upon reasonable request.
