# Supplementary material for: Engineering Resatorvid‐Loaded Sub‐Microgels of Epigallocatechin‐3‐gallate/Hyaluronic Acid to Treat Acute Lung Injury
Source: Adv Healthc Mater. 2026 Mar 24;15(21):e05916. doi: 10.1002/adhm.202505916 (PMC13241471; doi:10.1002/adhm.202505916)
Supplement: Supplementary file 1 — Supporting File: adhm71077‐sup‐0001‐SuppMat.docx. [file ADHM-15-0-s001.docx]

Supporting Information

**Engineering Resatorvid-Loaded Sub-Microgels of Epigallocatechin-3-gallate (EGCG)/Hyaluronic Acid for to Treat Acute Lung Injury**

*Bo Liu, Olawale Alimi Alimi, Benjamin Stearnes, Svetlana Romanova, Brady J Sillman, Mena* *Asha Krishnan, Kristina Bailey, Benson J Edagwa, Huanhuan Joyce Chen, Han-jun Wang*, Bin Duan**

B. Liu, O.A. Alimi, B. Stearnes, M. A. Krishnan, B. Duan

Mary & Dick Holland Regenerative Medicine Program, University of Nebraska Medical Center, Omaha, NE, 68198 USA

* E-mail: [hanjunwang@unmc.edu](mailto:hanjunwang@unmc.edu); [bin.duan@unmc.edu](mailto:bin.duan@unmc.edu)

B. Liu, O.A. Alimi, B. Stearnes, M. A. Krishnan, B. Duan

Division of Cardiovascular Medicine, Department of Internal Medicine, University of Nebraska Medical Center, Omaha, NE, 68198 USA

B. Duan

Department of Surgery, University of Nebraska Medical Center, Omaha, NE 68198, USA

B. Duan

Department of Mechanical and Materials Engineering, University of Nebraska Lincoln, Lincoln, NE 68588, USA

S. Romanova

Department of Pharmaceutical Sciences, University of Nebraska Medical Center, Omaha, NE, 68198, USA

K. Bailey

Division of Pulmonary, Critical Care & Sleep Medicine, Department of Internal Medicine, University of Nebraska Medical Center, Omaha, NE, 68198, USA

K. Bailey

VA Nebraska Western Iowa Healthcare System, Department of Medicine, Omaha NE 68105.

B. J. Sillman, B.J. Edagwa

Department of Pharmacology and Experimental Neuroscience, University of Nebraska Medical Center, Omaha, NE, 68198, USA

H.J Chen

Pritzker School of Molecular Engineering, University of Chicago, Chicago, IL 60637, USA

Ben May Department for Cancer Research, University of Chicago, Chicago, IL 60637, USA

H.J. Wang

Department of Anesthesiology, University of Nebraska Medical Center, Omaha, NE, 68198, USA


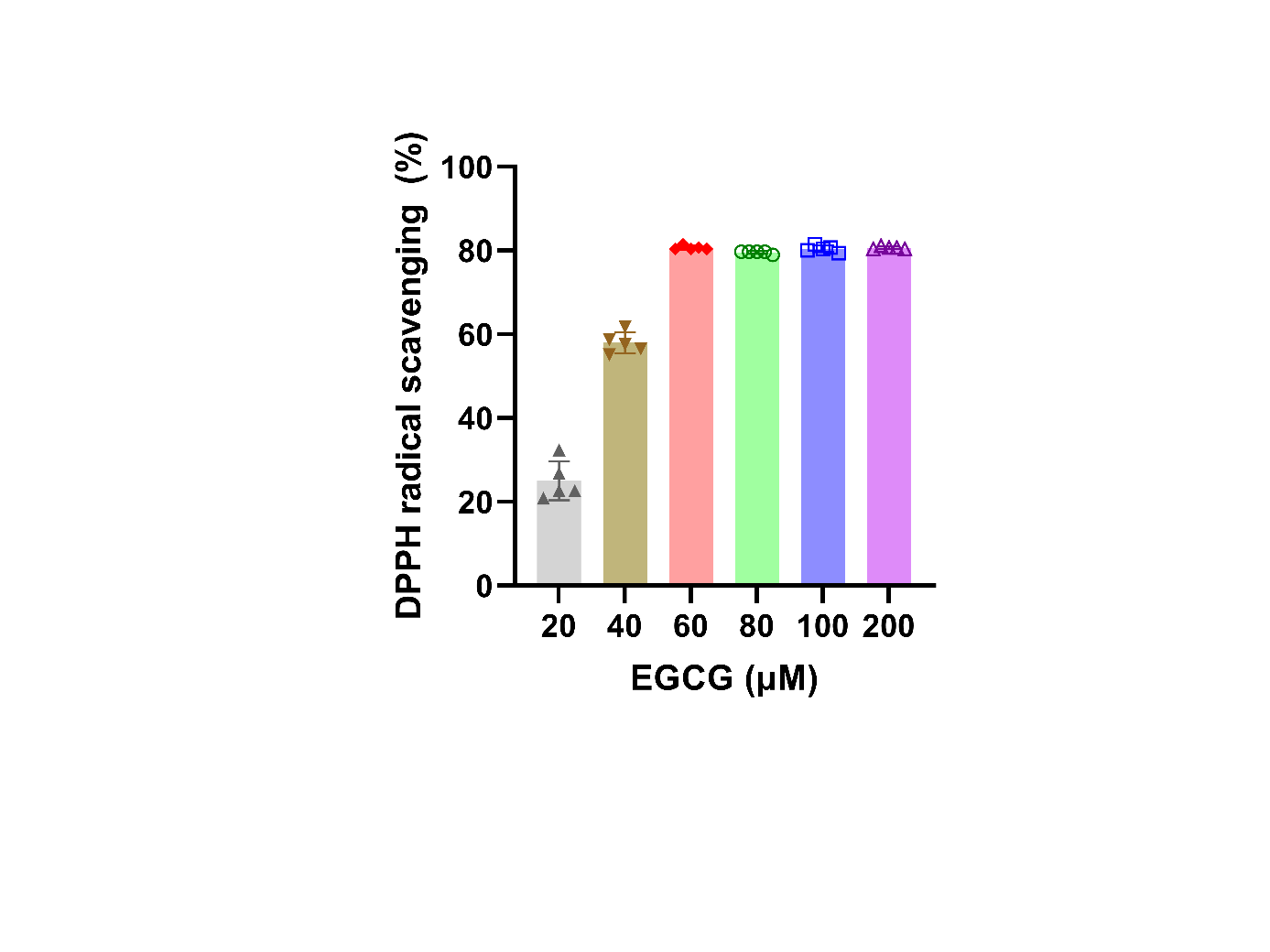


**Figure S1**. Analysis of the radical scavenging levels of EGCG by a DPPH assay.


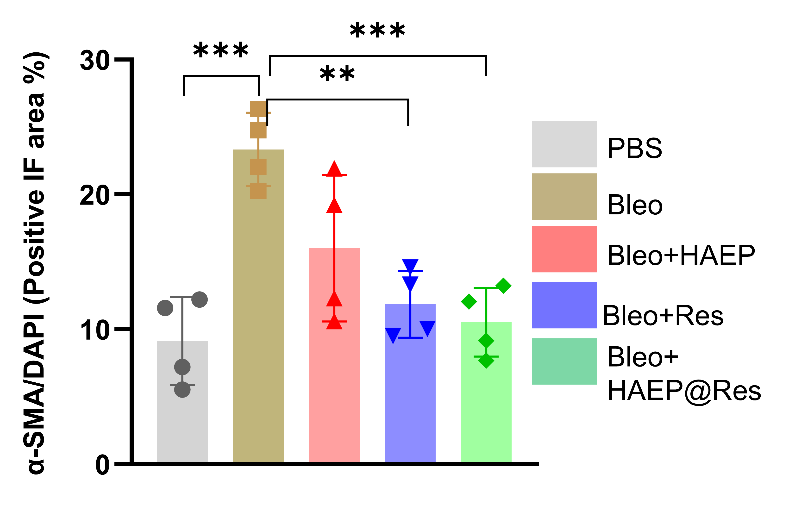


**Figure S2**. Semiquantitative analysis of the α-SMA positive area relative to the DAPI positive area. n=4; ***p* < 0.01; ****p* < 0.001.


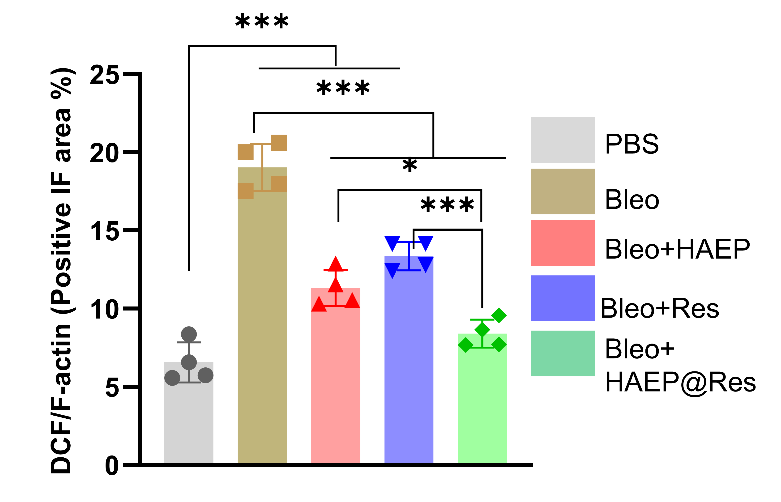


**Figure S3**. Semiquantitative analysis of the DCF positive area relative to the DAPI positive area. n=4; **p* < 0.05; ****p* < 0.001.

RAW264.7 cells were from American Type Culture Collection (ATCC) and cultured in RPMI 1640 medium (Gibco) supplemented with 10% FBS and 1% P/S at 37 ˚C, 5% CO_2_. To evaluate the effect of HAEP@Res on RAW264.7 against Bleo stimulation, 1 × 10^5^ RAW264.7 cells were seeded in each well of a 24-well plate. After incubation overnight, the cells were stimulated with 10ug/mL Bleo, and then treated with extracts media with Bleo of HAEP, and HAEP@Res (2 mg/mL). After being incubated for 1 day, the RAW264.7 were collected for qPCR quantitative analysis, including *Tnf*, *Il1b*, and *Ll6* genes. Negative control: RAW264.7 cells treated by PBS; Positive control: RAW264.6 treated by Bleo alone.

**
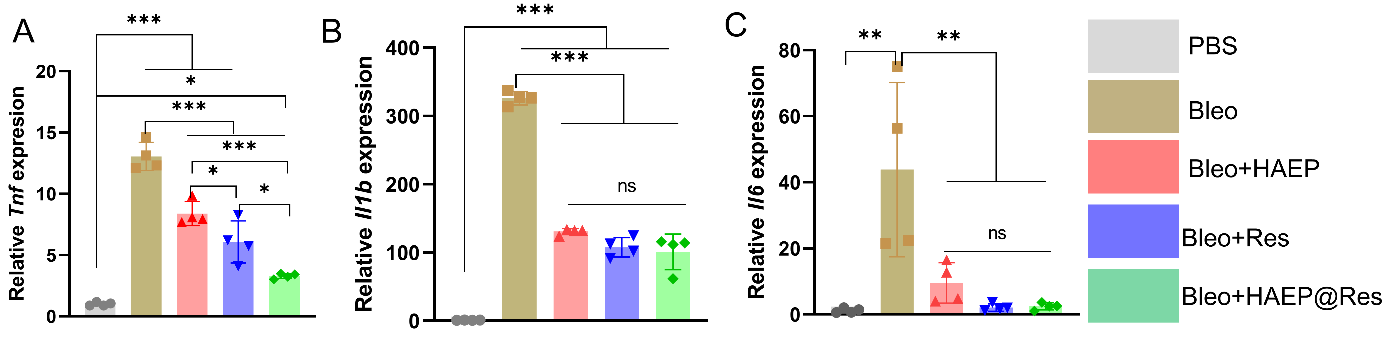
**

**Figure S4**. qPCR analysis of pro-inflammatory gene expression in RAW264.7 cells of different treatment groups *in vitro*: A: *Tnfα*; B: *Il1b*; C: *Il6*; n = 4, ns: no significance, **p* < 0.05; ***p* < 0.01; ****p* < 0.001.

*Immunofluorescence staining of α-SMA on lung tissues*: The isolated lung tissues were dissected and then fixed in 4% PFA solution and dehydrated in 30% sucrose. Then, the specimen was embedded in optimal cutting temperature (OCT) compound within a cryo-mold and cut into 12 μm-thick sections at −20 °C using a cryotome (CM1850, Leica). After being immerged in methanol for 5 min, all sections were permeabilized and blocked with 0.1% Triton X-100 and 5% goat serum in PBS for 2 h, the samples were incubated with α-SMA-Cy3 (C6918, Sigma) and F-actin phalloidin conjugates (ActinGrenn^TM^ 488, Invitrogen^TM^) at RT for 2 h and further incubated with DAPI (1: 1000, Invitrogen, D1306) for 30 min at RT. Finally, the stained samples were imaged with a confocal laser scanning microscopy (CLSM, Zeiss, LSM 800).


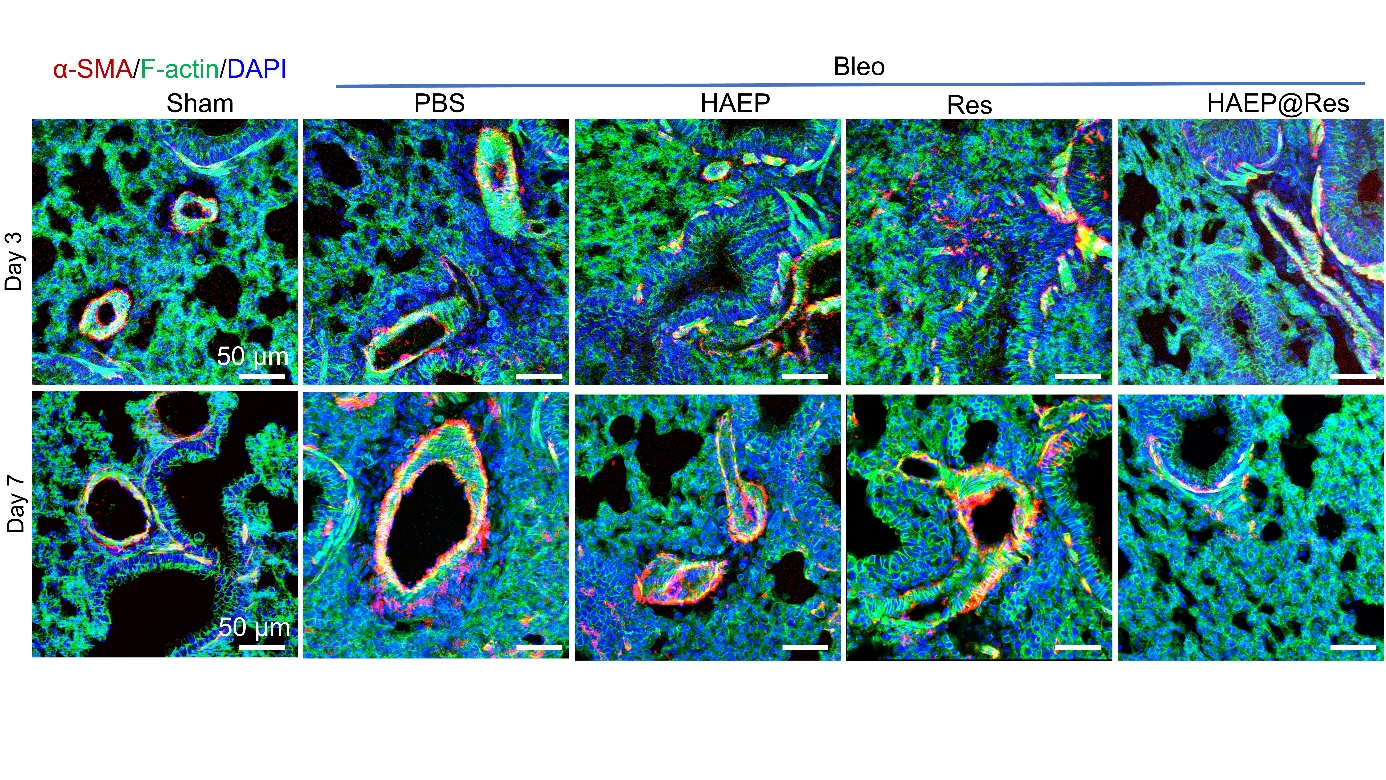


**Figure S5**. IF staining of CLSM images showing effect of HAEP@Res on attenuation of Bleo-induced α-SMA expression on day 3 and day 7 post-treatment.

**
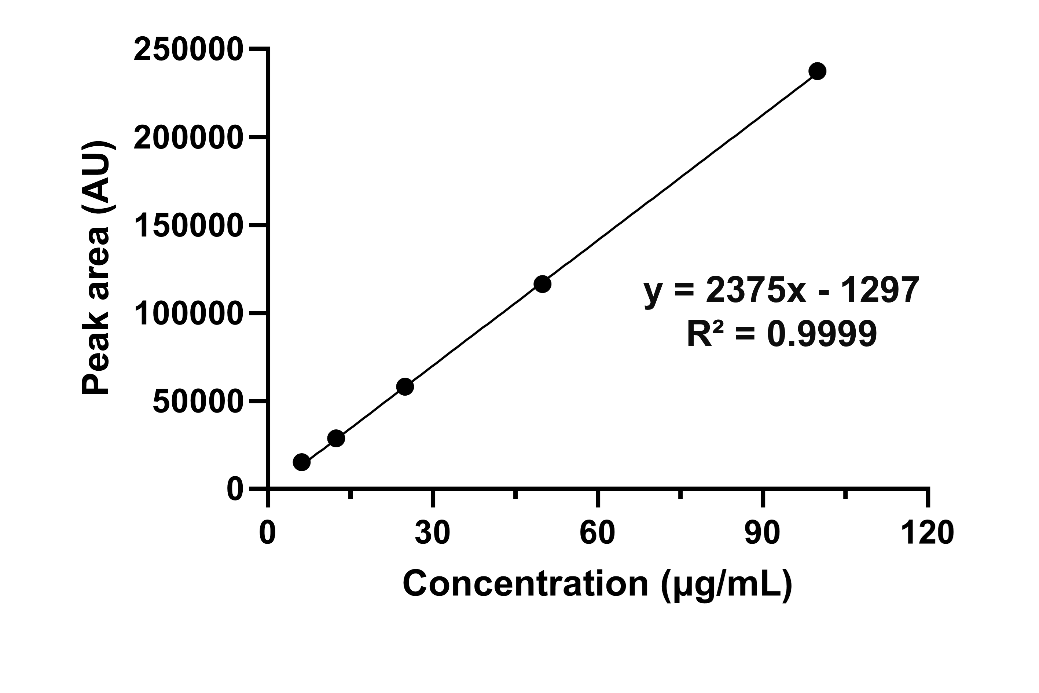
**

**Figure S6**. The standard curve of Res drug based on HPLC spectra.

**Table S1.** List of primers used for real-time qPCR in this work.

| Species | Gene name | Forward primer | Reverse primer |
| --- | --- | --- | --- |
| Mouse | *Gapdh* | CATCACTGCCACCCAGAAGACTG | ATGCCAGTGAGCTTCCCGTTCAG |
| Mouse | *Tlr4* | AGATCTGAGCTTCAACCCCTTG | GAGAGGTGGTGTAAGCCATGC |
| Mouse | *Tnf* | GAGTFACAAGCCTGTAGCCCA | TTGAGATCCATGCCGTTGGC |
| Mouse | *Il6* | AGACAAAGCCAGAGTCCTTCAGA | AGGAGAGCATTGGAAATTGGGG |
| Mouse | *Il1b* | TGCCACCTTTTGACAGTGATGA | TGCCTGCCTGAAGCTCTTGT |
| Mouse | *Nrf2* | CGCCCTCAGCATGATGGACT | TGTCTTGCCTCCAAAGGATGTCA |
| Mouse | *Nos2* | TTGGTGAAGGGACTGAGCTGTT | ACTTCCAGGGGCAAGCCAT |
| Mouse | *Tgfb* | CGCCTGAGTGGCTGTCTTTTG | TGGGGCTGATCCCGTTGAT |
| Mouse | *Ccl2* | GACCCCAAGAAGGAATGGGT | ACCTTAGGGCAGATGCAGTT |
